# Building Ventilation as an Effective Disease Intervention Strategy in a Dense Indoor Contact Network in an Ideal City

**DOI:** 10.1371/journal.pone.0162481

**Published:** 2016-09-09

**Authors:** Xiaolei Gao, Jianjian Wei, Hao Lei, Pengcheng Xu, Benjamin J. Cowling, Yuguo Li

**Affiliations:** 1 Department of Mechanical Engineering, The University of Hong Kong, Pokfulam Road, Hong Kong SAR, China; 2 Institute of Applied Mathematics, Academy of Mathematics and Systems Sciences, Chinese Academy of Sciences, Beijing, China; 3 WHO Collaborating Centre for Infectious Disease Epidemiology and Control, School of Public Health, The University of Hong Kong, Pokfulam Road, Hong Kong SAR, China; Columbia University, UNITED STATES

## Abstract

Emerging diseases may spread rapidly through dense and large urban contact networks, especially they are transmitted by the airborne route, before new vaccines can be made available. Airborne diseases may spread rapidly as people visit different indoor environments and are in frequent contact with others. We constructed a simple indoor contact model for an ideal city with 7 million people and 3 million indoor spaces, and estimated the probability and duration of contact between any two individuals during one day. To do this, we used data from actual censuses, social behavior surveys, building surveys, and ventilation measurements in Hong Kong to define eight population groups and seven indoor location groups. Our indoor contact model was integrated with an existing epidemiological Susceptible, Exposed, Infectious, and Recovered (SEIR) model to estimate disease spread and with the Wells-Riley equation to calculate local infection risks, resulting in an integrated indoor transmission network model. This model was used to estimate the probability of an infected individual infecting others in the city and to study the disease transmission dynamics. We predicted the infection probability of each sub-population under different ventilation systems in each location type in the case of a hypothetical airborne disease outbreak, which is assumed to have the same natural history and infectiousness as smallpox. We compared the effectiveness of controlling ventilation in each location type with other intervention strategies. We conclude that increasing building ventilation rates using methods such as natural ventilation in classrooms, offices, and homes is a relatively effective strategy for airborne diseases in a large city.

## Introduction

Infectious disease epidemics such as the 2003 SARS epidemic, the 2009 H1N1 pandemic, and the 2015 MERS epidemic are a threat to public health. More than 10% of annual deaths worldwide, i.e., 6 million deaths, are caused by viral or bacterial respiratory infections [1]. Due to increasing population density and a more connected world, new or non-vaccinable respiratory infectious diseases [2], especially airborne diseases [3–5], can be widely and rapidly transmitted.

Many interventions for controlling airborne disease transmission have been studied, such as masks, hand hygiene, isolation, quarantine, vaccination, school closure, etc. [6, 7]. Building ventilation is known to be effective for reducing the spread of airborne diseases such as tuberculosis, SARS, smallpox, chicken pox, and influenza in single indoor environments [8–12], but has not been studied at the community level. Ventilation intervention decreases transmission probability by directing the flow of airborne infectious agents away from susceptible persons and/or by removing infectious agents from room air. Because it relies less on individual compliance, ventilation has an advantage over other non-pharmaceutical interventions (e.g., hand washing or mask use). But questions remain. Is ventilation as effective at the community level as other interventions?

Airborne infection, if any, occurs predominantly in indoor environments, where people in modern society spend more than 90% of their time [13]. An individual infected in one building may infect others in the building(s) that he/she subsequently visits. People move from one indoor environment to another, and are in frequent contact with others, creating an indoor contact network in which an airborne disease can spread. To study ventilation as a community-level intervention, the disease spread dynamics must be ideally modeled at the individual person and location level for an entire urban social network [14]. Many widely used social contact network models, such as a random network [15] or a small world network [16, 17], may be too simplistic to describe the complicated dynamic between individuals and indoor environments. Environment parameters such as ventilation cannot be considered in these models, as in these models location is not refined to the individual indoor-space level. More recently, Eubank and colleagues [18, 19] developed a location-based social network model. However, the resources for building a realistic urban network in a large city are generally not available.

One of the significant limitations on the simulation of an indoor-contact network at the community level is computational power. In a large city such as Hong Kong, there are around 7 million people (*N*_*P*_) and an estimated 3 million indoor locations (*N*_*L*_, as a building can have many indoor locations). Ideally, we need to know the probability and duration of any two people meeting in any one indoor space in Hong Kong at any time. That is, we need to know “*Who meets whom*, *where*, *when*, *and for how long*.” Clearly, to model a large city there is need for a fast algorithm and huge computer storage capacity. The computer storage requirement can be formidably large; it is proportional to the cubic of the population ($N_{P}^{3}$), assuming that the indoor space population is at the same order of population as in Hong Kong. It is thus unrealistic to trace every individual on an hourly scale in a large city, i.e., when and where each person enters and leaves a room.

Consequently, we constructed a probability indoor contact model for an ideal city using real Hong Kong data with a linear memory requirement. Our model used the available data on population, buildings, and indoor spaces; the probability of people visiting each location type; ventilation rates; etc. Then, we developed a probability urban airborne transmission model by integrating the indoor contact network model with both the SEIR model for the spread of community diseases and the Wells-Riley equation for local infection risks. The transmission dynamics of a hypothetical airborne disease outbreak was studied and the effectiveness of different interventions such as isolation, home isolation, and increasing ventilation were evaluated and compared. In this feasibility study, we assumed that this hypothetical airborne disease has the same natural history and infectiousness as smallpox with no pre-existing immunity in the population.

## Methods

### Building an indoor contact network for an ideal large city

Indoor contact was defined as two or more individuals visiting the same indoor location at the same time. In other studies, contacts are often defined as close, conversational, or physical contact [20, 21]. Our indoor contact network model starts with the so-called individual/location approach, which has three stages.

#### Stage 1

Generating both people and indoor space populations. A synthetic population was first generated that includes every individual in Hong Kong. At the same time, a synthetic indoor space (location) was generated that includes every indoor space in Hong Kong. 6,857,100 people were divided into eight *population groups* (home stayers, office workers, classroom attendees, food service workers, shop workers, drivers, public space workers, and others), and 2,923,035 locations into seven *location groups* (home, office, classroom, restaurant, shop, transport vehicles, and other public locations). Each individual was assigned a unique person ID and an occupation (population group), and each location a unique location ID, a function (location group), a size, an occupant density, and a ventilation rate. The inclusion of ventilation rate as a characteristic of locations allows us to study for the first time the effect of ventilation on the spread of disease.

#### Stage 2

Defining the location visiting schedule and behavior of each individual. This is a crucial stage for tackling the formidably cubic computer storage requirement. To construct a simple probability indoor contact model with a linear memory requirement, we proposed an ideal city with the following three simplifications.

**Fig 1:**
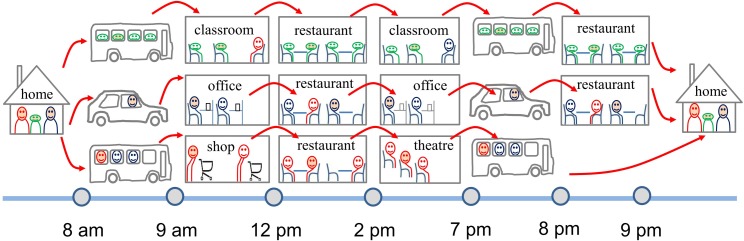
An example of hourly indoor activity sequences for a family of three individuals with different occupations. They meet other people in different types of locations (represented with different colors and shapes) during a typical workday. There are 6,857,100 people and 2,923,035 locations in Hong Kong.

First, each population group has a fixed daily schedule. Individuals in each population group have a fixed and identical location visiting schedule. All of the individuals in the entire population spend the same duration at any location in a location group. The location visiting schedule for every individual in a population group is defined, e.g., for office workers it is “home → transportation → office → lunch → office→ transportation → dinner → home” (see Fig 1). We denote *g* = 1,2,…, *G* as the sequence of location groups of individuals’ location visiting behavior during a simulation day. As we assume that all of the visitors enter and leave a location at the same time, we can simply define the meeting duration in a location group *g*, as *τ*_*g*_. We refer to this assumption as the “parallel session” approach, as in typical academic conferences.

Second, we assumed that any individual in a population group has the same number of *favorite locations* in each location group. For each location type, individuals choose a fixed number of favorite locations such as favorite shops, restaurants, etc., although offices and homes are unique to each individual. Individuals only visit their favorite locations. We use *l* = 1,2,…*L*_*g*_ to represent an individual’s collection of possible choices in each location group.

Third, the individual location visiting probability for locations in each location group was assumed the same for all individuals from the same population group. Hence, the probability of visiting a favorite location can be determined for each individual in each location group.

The first simplification deals with “when and how long,” and the second with “where.” These two simplifications allow us to use an array *η*_*j*,*l*_ (*l* = 1,2,…*L*_*g*_) to represent the probability of individual *j* visiting location *l*. As there are only *g* = 1,2,…, *G* location groups, the total storage requirement is reduced to a linear requirement *N*_*P*_ × *L*_*g*_ × *G*. This simplification allows us to handle a population of 7 million visiting 3 million locations on our desktop computers.

The third simplification implies that the location visiting behavior of individuals is determined solely by their occupations. McPherson et al. [22] demonstrated that social contact behavior is strongly related to the occupation and education level of individuals. Edmunds et al. [20] and Read et al. [23] also suggested that occupation is a primary influence on face-to-face contacts, especially during weekdays. The contact patterns and contact time are correlated with contact locations [23].

Hence, a pre-defined 24-hour activity sequence can be easily assigned to each synthetic individual, according to their profession and social duties. The time step for the activity sequence was chosen to be 30 minutes. Finer time steps can also be used, but are not necessary as the incubation period is relatively long.

Note that we do not associate each individual with other demographic attributes such as income level or age. In our simple model, each population group has the same location visiting schedule, and each location group has the same occupant density. Visiting probabilities for individuals in a population group to a type location were generated randomly assuming the population distribution follows a normal distribution (the mean is the location-visiting proportion and the standard deviation is10% of the mean value, see S1 File for detailed description). We used the available statistical data on population, buildings, and the probability of people visiting different indoor environments. For example, the probability that students will have lunch in a restaurant, or that a teacher will take a bus to go to work, were estimated according to the available data drawn from social behavior studies and household expenditure surveys.

#### Stage 3

**Fig 2:**
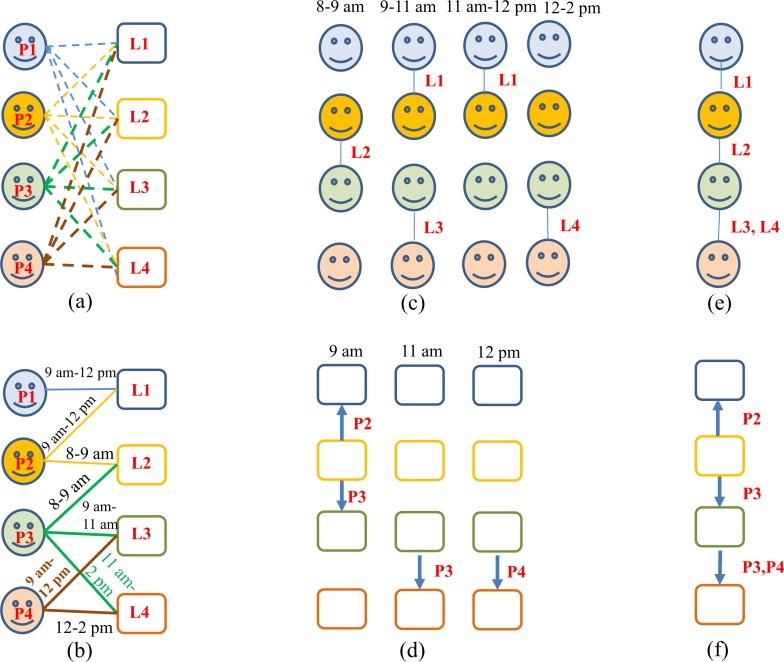
Revised Eubank’s bipartite graph of a small indoor contact network. **(a)** A bipartite graph of a probability network with two types of vertex and four people (represented with a p or a smiling face), and four indoor spaces (represented by l or an open square). P1 has a favorite location. Each person p can visit each location l with a given probability, so there is an edge in the graph between each p and each l. People vertices are labeled with occupation and favorite locations. Location vertices are labeled with type, floor area, and ventilation rate. Edges are labeled with arrival and departure times, and visiting probability. **(b)** A bipartite graph of a fixed network, which is otherwise the same as (**a)**, but the edges are labeled with arrival and departure times. **(c, d)** The two disconnected graphs Gp and Gl induced by connecting vertices that were separated by exactly two edges in Gpl. **(e, f)**. The static projections resulting from ignoring time labels in Gp and Gl.

Constructing the indoor contact network (people-location *probability* network, ${\bar{G}}_{I,L}$). For our approach, a probability network is preferred, given the uncertainty of individual daily routes. In this people-location *probability* network, all of the individuals are connected through their favorite locations, see Fig 2. Both individuals and locations are represented by nodes. An edge between an individual node and a location node denotes a connection between the individual and location (i.e., a location visiting). The weight of the edge represents the *probability* that the individual visits the location *in a day*. In implementation, location nodes were selected to connect each individual with their favorite locations, and the edges were weighted according to the probability that the individual will visit the location group. A recursion algorithm was used to ensure that the occupants of all of the locations satisfy their limits.

Detailed descriptions of the data collection process and the indoor contact network construction methodologies are given in S1 File. In reality, each individual in the network might connect with many other locations with different edge weights. A real network is also dynamic with some edges changing periodically (e.g., weekly or seasonal changes) or permanently. However, it is in practice impossible to describe mathematically these complicated features of the network. Here, we neglected weekly or seasonal changes when calculating ${\bar{G}}_{I,L}$.

### Integrating the indoor contact network model with the Wells-Riley equation

The airborne transmission risk is estimated using the Wells-Riley equation [24]. In an enclosed indoor environment, infectious airborne droplet nuclei are assumed to be evenly dispersed. Let the probability of susceptible individual *i* being infected by the only infector *j* in indoor environment *l* be *p*_*i*,*j*,*l*_, which can be expressed by the following equation [24]:

$$P_{i,j,l}=1-\exp(-\frac{pQ\tau_{l}}{q_{l}}),$$ (1)

where *Q* is the average number of infectious quanta generated by a infector (quanta/h); *p* is the pulmonary ventilation rate, and here we use 0.38 m^3^/h [25]; *τ*_*l*_ is the duration of exposure to infection (h); and *q*_*l*_ is the ventilation rate of the location (m^3^/h). The infection bears a Poisson relation to the dose of inhaled infective agents (droplets or droplet nuclei), according to Wells [24].

One possible solution for integration is to generate the social contact network from the people-location probability network, and simulate the disease’s dynamic with the Markov chain Monte Carlo (MCMC) method. However, this method is too time-consuming for a large population. Hence, we simulated the *epidemic* as a Markov chain with each simulation day as a time step. We defined the infection states of individuals as probabilities. The calculated local infection risk of an individual in a contaminated location using the Wells-Riley equation becomes a joint probability.

Let $I_{j}^{t}$ be the probability of individual *j* being an infector on day *t*, and *η*_*j*,*l*_ represent the probability of individual *j* visiting location *l*. The visiting probability of an individual to a location is assumed to be independent of other individuals’ choices. In the meantime, the visiting probabilities within the set of favorite locations are given, as the indoor contact network is built. Therefore, the location visiting probability of individual *i* to location *l* is independent of the other probabilities, including the probability of infection. This means that $I_{j}^{t}$ and *η*_*j*,*l*_ are independent, and hence the probability of individual *i* being infected by individual *j* in location *l* in the *g*^th^ location group in day *t*, $a_{i,j,l}^{t,g}$, can be calculated as

$$a_{i,j,l}^{t,g}=P_{i,j,l}\eta_{j,l}I_{j}^{t}.$$ (2)

The visiting time of any individual to a given location is identical, according to the following equation:

$$a_{i,j,l}^{t,g}=(1-\exp(-\frac{pQ\tau_{g}}{q_{l}}))\eta_{j,l}I_{j}^{t}.$$ (3)

The probabilities of individual *i* being infected from different infectors in location *l* can be assumed to be independent. This is not difficult to understand, as a quanta is defined as a dose of infective agents that, when inhaled, can cause an infection. Assuming the concentration of each quanta is constant and well-mixed, the event of breathing in a quanta became a Poisson process [23]. Hence, the probability of individual *i* being infected in location *l* can be calculated as

$$b_{i,l}^{t,g}=1-\prod_{j=1}^{N_{l}}\left(1-a_{i,j,l}^{t,g}\right).$$ (4)

Here, *N*_*l*_ is the number of possible visitors to location *l*. Assuming there are $L_{i}^{g}$ favorite locations in the *g*^th^ location group, we mark them as $l=1,2,\cdots ,L_{l}^{g}$. It is assumed that the infection risks from the favorite locations that an individual may choose in a visiting step are non-correlated. As individual *i* can only be in one location at a time and has a given probability of being infected in the selected location, the infection probabilities of individual *i* for different locations in the same location group are mutually exclusive. Thus, we can calculate the infection probability for individual *i* in the location group $c_{i}^{t,g}$, as

$$c_{i}^{t,g}=\sum_{l=1}^{L_{i}^{g}}b_{i,l}^{t,g}\eta_{i,l}.$$ (5)

$S_{i}^{t-1}$ is the probability of individual *i* being susceptible to infection on simulation day *t* − 1. As the daily schedule of an individual’s location visiting behavior is given, for each step of the individual’s movement, the infection stage of the individual can be assumed to correlate only with the previous step. Hence, we use $d_{i}^{t,g}$ to demonstrate the susceptibility of an individual after visiting group *g* as follows:

$$\{\begin{array}{c} d_{i}^{t,g}=(1-c_{i}^{t,g})S_{i}^{t}\;g=1\\ d_{i}^{t,g}=(1-c_{i}^{t,g})d_{i}^{t,g-1}\;g=2,\cdots ,G \end{array}.$$ (6)

Then,

$$S_{i}^{t}=d_{i}^{t,G}=S_{i}^{t-1}\prod_{g=1}^{G}\left(1-c_{i}^{t,g}\right).$$ (7)

According to Eqs (3), (4), (5) and (7),

$$S_{i}^{t}=S_{i}^{t-1}\prod_{g=1}^{G}\left(1-\sum_{l=1}^{L_{i}^{g}}\eta_{i,l}(1-\prod_{j=1}^{N_{l}}(1-(1-\exp(-\frac{pQ\tau_{g}}{q_{l}}))\eta_{j,l}I_{j}^{t})\right)).$$ (8)

**Fig 3:**
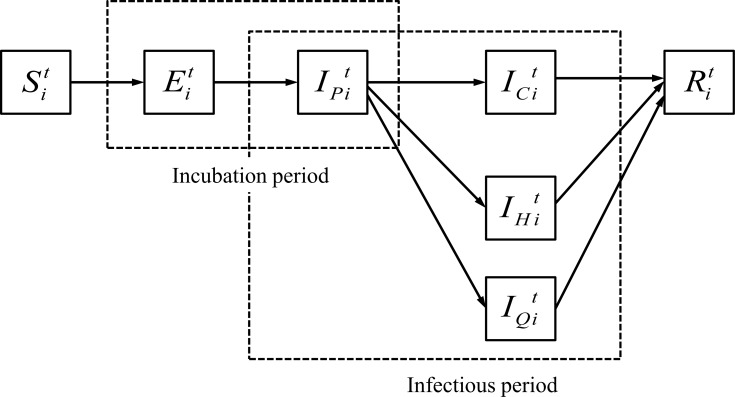
The natural history of the airborne disease infection. We assume that in the natural history of airborne diseases a susceptible individual will pass through a latent period ($E_{i}^{t}$, 11.6 days) after being exposed, followed by a symptomatic state ($I_{Pi}^{\;t}$, 2.49 days), and will eventually reach an infectious state during which a rash appears ($I_{Ci}^{\;t}$, 18.3 days). Both the fever onset and rash states are considered infectious [26]. Infected individuals will either recover or die ($R_{i}^{t}$). We assume that in the baseline outbreak scenario, 50% of the patients with a rash will be hospitalized and isolated ($I_{Hi}^{\;t}$), 95% of the rest will be isolated at home ($I_{Qi}^{\;t}$); during the latter period the individual will stay home and not visit any other locations.

In the sketch of the natural history of the airborne disease (the same as smallpox), shown in Fig 3, each state of an individual’s infection is represented by the probability of this event. A susceptible individual *i*, after exposure, will pass through a latent period ($E_{i}^{t}$) before infection, followed by a pre-symptomatic infection period ($I_{Pi}^{\;t}$), eventually reaching an infectious state. Patients with severe symptoms will be hospitalized and isolated ($I_{Hi}^{\;t}$), and people with symptoms will comply with home isolation ($I_{Qi}^{\;t}$). The death rate is not considered in this case.

**Fig 4:**
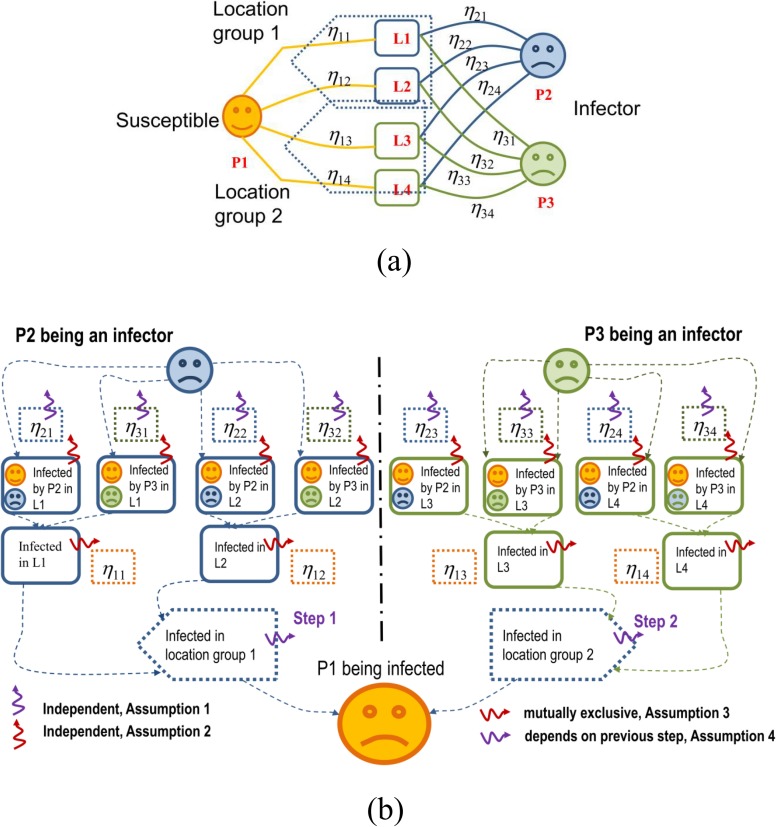
Calculation of transmission probability based on different assumptions. **A**. A simple example of the location visiting behavior of A, B, and C. Individual A is susceptible to infection and individuals B and C both have the probability of being an infector. Each of the three individuals has two location visiting steps in their daily schedule. In each simulation day they can choose to visit either location J or K from group 1 in the first step, and location H or M from group 2 in the second step. The location visiting probability of individual A to location J is defined as *η*_*A*,*J*_. **B**. The diagrammatic flow chart of the method for calculating infection risks from individuals B and C to individual A (Solid arrow) and the correlations of variables (dashed arrow) given by Assumptions 1 to 4.

A simple case of three individuals visiting four locations in two location groups is shown in Fig 4.

The epidemic was modeled discretely with a stated time step of one day. The state of each individual in the infection-recovery process in the current time step depends on their state in the previous time step. Hence, the transmission process becomes a Markov chain. The Markov chain transmission probability for a single individual can be represented by the following equations:

$$\left\{ \begin{array}{l} E_{i}^{t}=E_{i}^{t-1}+S_{i}^{t}-S_{i}^{t-1}-\theta E_{i}^{t-1}\\ I_{Pi}^{\;t}=I_{Pi}^{\;t-1}+\theta E_{i}^{t-1}-\phi I_{Pi}^{\;t-1}\\ I_{Ci}^{\;t}=I_{Ci}^{\;t-1}+(1-\varpi -\vartheta )\phi I_{Pi}^{\;t-1}-\frac{1}{\left(1/\gamma -1/\phi \right)}I_{Ci}^{\;t-1}\\ I_{Hi}^{\;t}=I_{Hi}^{\;t-1}+\varpi \phi I_{Pi}^{\;t-1}-\frac{1}{\left(1/\gamma -1/\phi \right)}I_{Hi}^{\;t-1}\\ I_{Qi}^{\;t}=I_{Hi}^{\;t-1}+\vartheta \phi I_{Pi}^{\;t-1}-\frac{1}{\left(1/\gamma -1/\phi \right)}I_{Qi}^{\;t-1}\\ R_{i}^{t}=R_{i}^{t-1}+\frac{1}{\left(1/\gamma -1/\phi \right)}(I_{Ci}^{\;t-1}+I_{Hi}^{\;t-1}+I_{Qi}^{\;t-1}) \end{array}\right.,$$ (9)

where $S_{i}^{t}$, $E_{i}^{t}$, $I_{Pi}^{\;t}$, $I_{Ci}^{\;t}$, $I_{Hi}^{\;t}$, $I_{Qi}^{\;t}$, and $R_{i}^{t}$ are the probabilities that individual *i* on day *t* is, respectively, susceptible, exposed (latent), pre-symptomatic, symptomatic infectious, hospitalized, home-isolated, and recovered or removed from the social network at day *t*. 1 / *θ*, 1 / *ϕ*, and 1 / *γ* are the latent, pre-symptomatic infectious, and total infectious period. *ϖ* and *ϑ* are the hospitalization rate and home isolation rate. According to Eq (8) we have

$$S_{i}^{t}=S_{i}^{t-1}\prod_{g=1}^{G}(1-\sum_{l=1}^{L_{i}^{g}}\eta_{i,l}(1-\prod_{j=1}^{N_{l}}\left(1-(1-e^{-\frac{pQ\tau_{g}}{q_{l}}})(I_{Pj}^{\;t-1}\eta_{j,l}+I_{Cj}^{\;t-1}\eta_{j,l}+I_{Qj}^{\;t-1}\eta_{j,l}^{\prime })))\right),$$ (10)

where $\eta_{j,l}^{\prime }$ is the location visiting probability after home isolation. If *l* is the home of *j*, $\eta_{j,l}^{\prime }=1$, otherwise $\eta_{j,l}^{\prime }=0$.

Eq (10) is referred to as the integrated indoor contact network and disease transmission model. It uses the probabilities of individuals *i* and *j* visiting location *l*, that is *η*_*i*,*l*_, and *η*_*j*,*l*_, from the probability indoor contact network model.

### Input parameters

To test the indoor contact network model, statistical data describing population groups and location groups were collected from the Hong Kong Census and Statistics Department and other related studies. The ventilation rates of the locations were estimated by reviewing relevant field measurements conducted in Hong Kong (see S1 File). The baseline ventilation rates were set as: 0.7 air change per hour (ACH) in homes, 2 ACH in classrooms, 1 ACH in offices, 1 ACH in restaurants, 1 ACH in shops, 4 ACH in transportations, and 1.4 ACH in other public locations. Unlike the agent-based approach of Eubank et al. [18], the input data for our simple network model are minimal. We are not interested in the exact choices of activity, travel mode, departure and estimation (and or time) choice, car following, etc.

One major difficulty in using the Wells-Riley equation for our purpose is obtaining a meaningful quanta generation rate. It may be estimated from reported outbreaks, but for some diseases the estimated values of different outbreaks are significantly different [10, 12, 25, 27–29]. Such significant variations may be due to differences in the sub-strains of the viruses, respiratory activities (number and size distribution of droplets or droplet nuclei generated from the index patient) [30, 31], the state of infection of the source patients [32], possible existence of super-spreaders [33], environment conditions such as temperature or humidity that relate to virus or bacterial survival rate [34], and significant uncertainties in the estimated ventilation condition [28]. Therefore, the estimated quanta generation rate from reported outbreaks may not be suitable for analyzing population transmission dynamics.

In intervention studies, the basic reproductive number, *R*_*o*_, is commonly used as an indicator of the transmission ability of the infection [35]. The transmission (diffusion) of diseases through a social network is generally estimated using the effective reproductive number. The correlation of quanta generation rate and *R*_*o*_ can be calculated for a single room outbreak by integrating the Wells-Riley equation and the compartment model [28]. This inspired us to propose an alternative approach for obtaining the quanta generation rate. We assumed that infectors have a constant quanta generation rate in the disease outbreak. We modeled such an outbreak in Hong Kong by assuming that the disease is transmitted by the airborne route only. The simulations were carried out with a range of quanta generation rates of 1, 2 or 3 quanta/h. The basic reproductive number was calculated for each simulation, with a respectively value of 2.22, 4.03 and 5.83 A correlation was then established between the basic reproductive numbers and the quanta generation rates Q.

The range of the estimated basic reproductive numbers agrees with the commonly adopted values of smallpox transmission, which are in the range of 3.5 to 10 [26, 35, 36]. Hence, we adopted three cases representing low, medium, and high transmission rate scenarios.

## Results

### Structure of the indoor contact network

**Fig 5:**
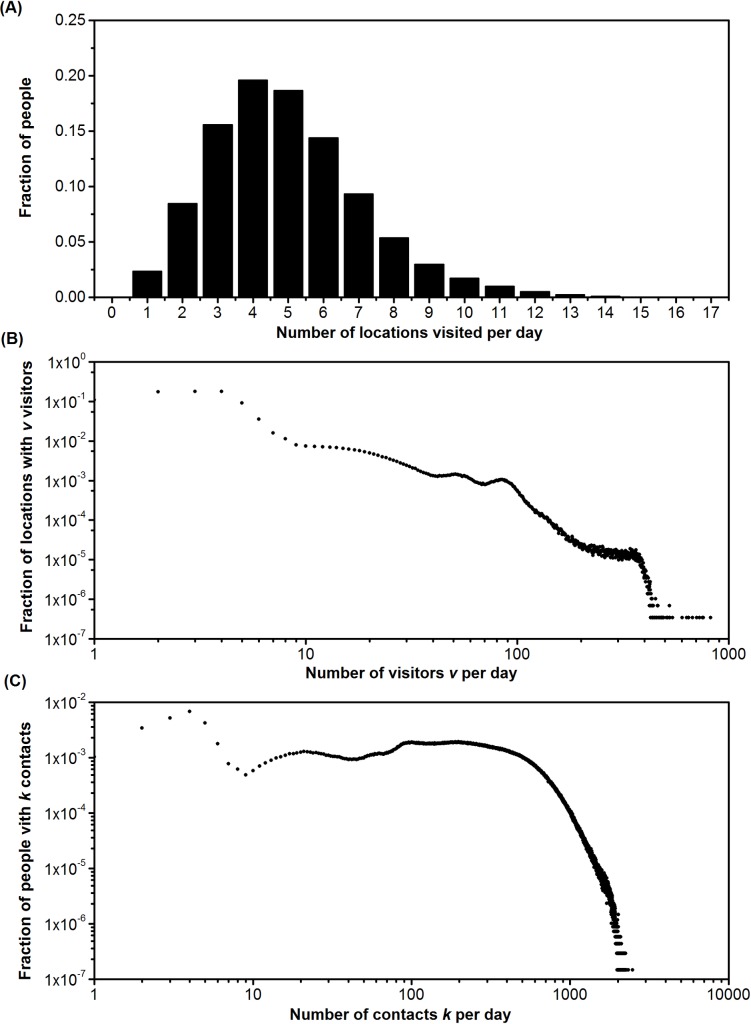
Degree distributions of people-location graph. **A**. Degree distribution of individuals with different numbers of daily visiting locations. **B**. Degree distribution of locations with different numbers of visitors (per day) on a log-log scale. **C**. Degree distributions of people-people graph on a log-log scale. There are 6,857,100 people and 2,923,035 locations in Hong Kong.

Our model produced a bipartite graph with two types of vertices, people and location. In our case, all of the locations are indoor spaces. People are connected by location (indoor spaces), and indoor spaces are connected by people. To understand the basic feature of the network structure, we generated a fixed network *G*_*I*,*I*_, based on a people-location probability network ${\bar{G}}_{I,L}$, using random numbers to generate fixed edges between connected individual nodes and location nodes in network ${\bar{G}}_{I,L}$. The degree distribution of *G*_*I*,*L*_ is plotted in Fig 5B. The location degree distribution of graph *G*_*I*,*L*_ is better fitted to the power-law distribution than the population degrees distribution of graph *G*_*I*,*I*_, which agrees with the findings of Eubank et al. [18]. The location graph is scale-free, meaning that a few locations (hubs) have many “visitors,” but the majority of locations have relatively few “visitors.” Eubank et al. argued that removing locations with more than 100 degrees (visitors) may improve the results. Such a network structure can be revealing in terms of infection control [18], e.g., locations of over 100 degrees need better ventilation.

The people-people graph *G*_*I*,*I*_ can then be generated by connecting individuals who are linked by common locations based on *G*_*I*,*L*_ with degree distribution; the result is shown in Fig 5C. The people contact graph is like a small-world, highly connected (not scale-free) network. Eubank et al. [18] suggested that vaccinating long-distance travelers is crucial for infection control. Our study cannot validate this conclusion, as we do not model travel distance. However, as Hong Kong has effective public transport and a small land area (less than 1000 km^2^), most of the locations visited by an individual are within one-hour travel. The people-people graph fits reasonably well with the predictions in [18]. The noise of two degree distributions might have been introduced by the compartmentalization of population and locations.

### A hypothetical airborne disease outbreak under the baseline scenario

**Fig 6:**
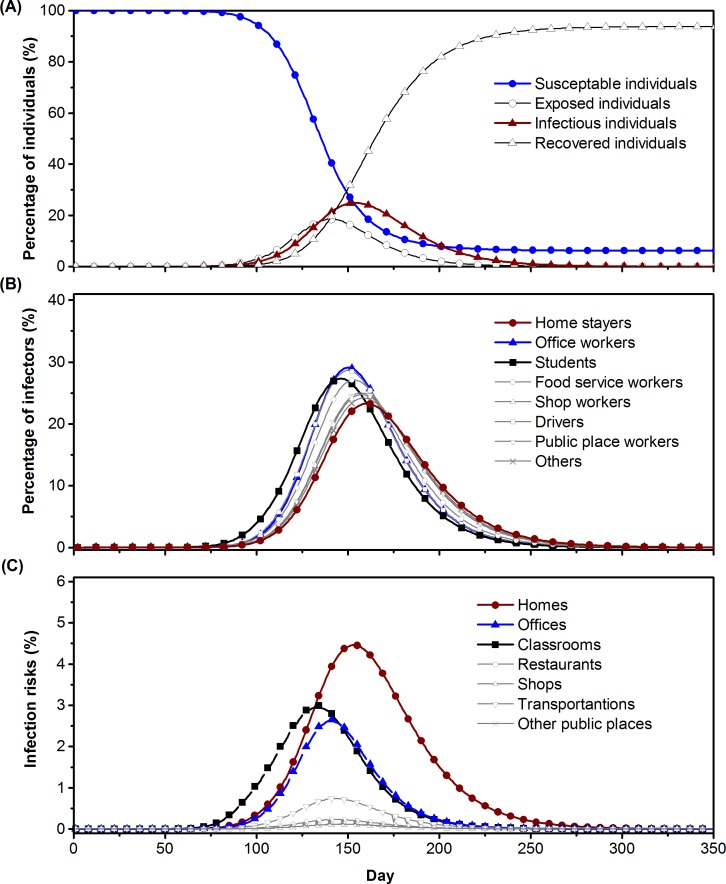
Outbreak dynamics of a hypothetical airborne disease outbreak in Hong Kong under the baseline scenario. A. The expected percentages of susceptible, exposed, infectious, and recovered individuals. B. The expected percentages of infectors in each population group. C. Average infection risks exposed in locations in different location groups.

In this study, we assume the complete susceptibility of the whole population. In the baseline outbreak scenario, the air change rates of different types of locations are set to be consistent with data from investigations of ventilation rates (see S1 File). The baseline outbreak case is simulated based on the indoor contact model without any interventions. We present the epi-curve of the outbreak, infection of individuals in different population groups, and infection risk in different types of locations in Fig 6.

The changes in the daily numbers of individuals in each infection state are plotted in Fig 6A. The peak of infection in the community occurs 154 days after the first infector is introduced into the community. Most of the individuals in the community are infected and the outbreak lasts for about 500 days. According to Fig 6B, the rate of infection among students rises first, but the number of infectors is highest in offices workers and lowest in home stayers.

Among the different locations, the highest infection risks are in homes, followed by classrooms and offices. The infection risk for individuals working in restaurants, shops, public places, and transportation systems are not listed here, as the data in Fig 6C were calculated using the exposure time of the general population rather than those who work in these locations.

### Effects of increasing ventilation rates under the medium transmission scenario

**Fig 7:**
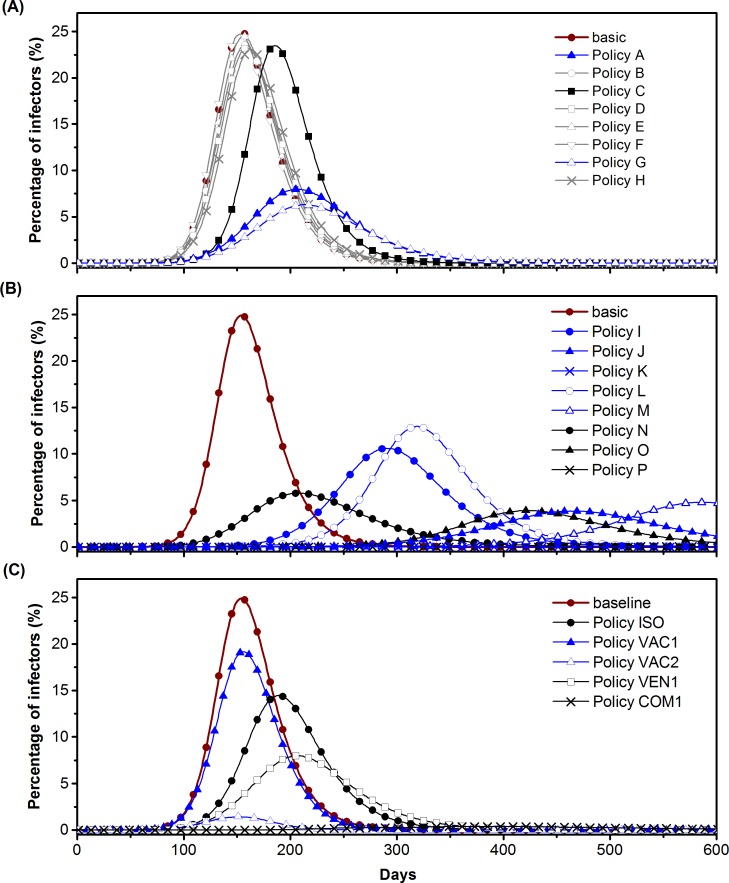
Control effects of increasing ventilation rates, isolation, and vaccination under a quanta generation rate of 2 quanta/h. A. Daily percentages of infectors under the baseline condition and control policies of increasing the ventilation rate in one type of location (Policy A to H). B. Baseline condition and increasing ventilation rate in all locations or a few types of locations (Policy I to P). The curves for Policy K and P are flat. C. Different ventilation, isolation, and vaccination control methods. The curves for Policy VAC3, VEN2, VEN3, and COM2 are all nearly flat (not shown).

The effects under different control methods are plotted in Fig 7. The attack rate, *δ*, time of peak infection, *T*_*p*_ (day), and percentage of peak infection, *λ*_*p*_, under Policies A to P are listed in Table 1. The control effects of increasing ventilation rates in one type of location (Policies A to H) under the medium transmission condition are shown in Fig 7A. Increasing the ventilation rate to 5 ACH (Policy A) or 10 ACH (Policy G) in all homes is an effective method for reducing the epidemic, and it reduced the attack rate substantially (around 50%). However, the differences between the two policies are not significant. Increasing the ventilation rate of all offices from 1 ACH to 5 ACH delays the arrival of peak infection, but it does not reduce much of the overall attack rate.

**Table 1:** Different ventilation control methods and their effects under a quanta generation rate of 2 quanta/h.

Policy	Policy content	Attack rate, *δ*	Time of peak infection, *T*_*p*_ (day)	Percentage of peak infection, *λ*_*p*_
Baseline		93.76%	154	24.90%
Policy A^1^	Homes, 0.7➔5 ACH	52.05%	206	7.97%
Policy B	Classrooms, 2➔5 ACH	93.19%	158	23.54%
Policy C	Offices, 1➔5 ACH	92.63%	186	23.47%
Policy D	Restaurants, 15 ACH	92.80%	159	23.22%
Policy E	Shops, 1➔5 ACH	93.48%	156	24.48%
Policy F	Public locations, 1.4➔5 ACH	93.62%	155	25.63%
Policy G	Homes, 0.7➔10 ACH	43.56%	211	6.30%
Policy H	Transportation, 4➔10 ACH	92.76%	163	23.07%
Policy I	All locations, double	73.06%	290	10.61%
Policy J	Transportation, 4 ACH	43.11%	464	3.86%
	Other locations, 3 ACH			
Policy K	All locations, 5 ACH	0.28%	1989	<0.01%
Policy L	All locations, 8 l/(s·person)	80.50%	319	12.95%
Policy M	All locations, 12 l/(s·person)	55.32%	587	4.79%
Policy N	Homes and classrooms, 5 ACH	43.49%	209	5.77%
Policy O	Homes and offices, 5 ACH	37.36%	426	3.89%
Policy P	Homes, classrooms and offices, 5 ACH	19.33%	1198	0.67%

^1^Policy A (Homes, 0.75 ACH) means increasing ventilation rate in all homes from 0.7 ACH to 5 ACH.

Increasing ventilation rates in all locations, demonstrated in Fig 7B, is more effective in delaying the peak infection than ventilation control in any one type of location, as it reduces the rapid transmission of the disease in large hubs at the very beginning of the outbreak. The policies of doubling ventilation rates (Policy I), applying 8 liters per second per person in all locations (Policy L), and applying 12 liters per second per person in all locations (Policy M) are all more effective than increasing the household ventilation rate to 5 ACH (about 85 liter per second per person; see S1 File). Increasing the ventilation rate in homes and offices largely delays and reduces the peak infection and has a control effect similar to that of a policy of applying 3 ACH in all locations (Policy J). Applying 5 ACH in all locations (Policy K) will diminish the outbreak.

### Comparison of effects of increasing ventilation rates with isolation and mass-vaccination

When the quanta generation rate is 2 quanta/h, the epi-curves of the daily percentages of infectors under different control policies (Table 2) are as plotted in Fig 7C. The control effects of all of the methods under different quanta generation rates are given in Table 2.

**Table 2:** Effects of isolation, vaccination, and ventilation on attack rate (*δ*), day of peak infection (*T*_*p*_), and percentage of infectors on day *T*_*p*_ (*λ*_*p*_) under different quanta generation rates.

Policy	Low transmission rate (1 quanta/h)			Medium transmission rate (2 quanta/h)			High transmission rate (3 quanta/h)		
	*δ* (%)	*T*_*p*_	*λ*_*p*_ (%)	*δ* (%)	*T*_*p*_	*λ*_*p*_ (%)	*δ* (%)	*T*_*p*_	*λ*_*p*_ (%)
Baseline	70.83	303	9.59	93.76	154	24.90	97.47	113	32.70
Policy ISO (Isolation) ^1^	1.15	897	0.04	72.34	190	14.47	79.48	203	16.51
Policy VAC1 (Vaccinating 10,000 people per day) ^2^	23.23	323	2.43	74.58	156	19.18	84.56	116	28.25
Policy VAC2 (50,000 people per day)	0.02	129	<0.01	6.00	153	1.40	34.24	121	10.78
Policy VAC3 (100,000 people per day)	Diminished			Diminished			2.72	100	0.94
Policy VEN1 (Homes, 5 ACH)	9.01	365	0.58	52.05	206	7.97	73.10	148	16.37
Policy VEN2 (Homes, offices and classrooms, 5 ACH)	Diminished			19.33	1198	0.67	60.53	372	7.88
Policy VEN3 (All locations, 5 ACH)	Diminished			0.28	1989	<0.01	33.63	554	2.69
Policy COM1 (ISO + VEN1)	0.16	1462	<0.01	5.63	372	0.37	24.81	281	2.46
Policy COM2 (ISO + VEN2)	Diminished			Diminished			Diminished		

^1.^ Isolation of 90% of infectors with rash, home isolation of 50% of fever patients

^2.^ Mass-vaccination given 20 days after the first infector is introduced to the model. The protect rate of the vaccination is assumed to be 95% [26]. The percentage of individuals accepting vaccination is assumed to be 80%.

The ability to control the epidemic with isolation is limited in the medium and high transmission scenarios, even though 90% of the infectors with rashes are isolated (13% of the population at the peak state, 2 quant/h) and 50% of the infectors with pre-symptomatic transmission are isolated in their households (5% of the population at the peak state, 2 quant/h). Also, the effect of mass-vaccination depends on the ability to distribute vaccinations. Vaccinating 100,000 susceptible individuals per day can diminish the infection in all three cases, whereas giving 10,000 vaccinations per day can only diminish the outbreak under the low transmission scenario; it leads to a reduction of less than 20% in the attack rates in the medium and high transmission scenarios.

Table 2 shows that higher ventilation rates can significantly reduce the disease transmission; in particular, it can delay and reduce the peak infection. Increasing the household ventilation rate (Policy VEN1) can mostly eliminate an outbreak under the low transmission rate, and is more effective than isolation (Policy ISO) or vaccination (Policy VAC1) under the medium and high transmission rate conditions. Applying isolation and increasing the household ventilation rates together (Policy COM1) largely restricts the outbreak in all three cases, and the number of isolated individuals is also 50 times smaller (less than 0.4% of population) than when isolation is applied alone in the medium transmission scenario.

## Discussion

### Ventilation is an effective intervention, particularly when the transmission rate is high

In this study, when the disease is completely airborne transmitted, increasing ventilation rates has demonstrated similar or even better control effects than isolation and vaccination. Furthermore, ventilation rates in homes, classrooms, and offices can reach up to 5 ACH simply by opening windows [37] or through the proper design of ventilation systems [38, 39], which is easier, more convenient, and more economical than isolating 12% of the population or mass-vaccination. Additionally, in the low transmission rate condition, both isolation and vaccination have acceptable control effects.

However, when the transmission rate is high, infection spreads rapidly before vaccinations can be distributed and the existence of the pre-symptomatic transmission limits the effect of isolation. In this case, combining increased ventilation rates with isolation has the best control effect and decreases the load of isolation requirements.

### Increasing ventilation in critical locations is important

Our comparison of increasing ventilation rate policies suggests that it is essential to apply targeted control strategies to the high infection risk locations. Homes are the most important locations for controlling infection throughout the community, due to longer exposure periods, household isolation of infectors, and most importantly the role of homes in connecting the infection transmission network. The ventilation rate can be readily increased to 5 ACH taking use of natural ventilation [40, 41].

In contrast, classrooms and offices play a role in connecting small hubs (homes) with medium and large hubs in the network. Infection in classrooms develops in the early stages, as shown in Fig 6, perhaps because of the indoor social contact networks and the ventilation rate settings in classrooms. In all classrooms, if an infector is introduced to the classroom, the rest of the students will develop infection rapidly (due to the long exposure time and low ventilation rate) and the transmission conditions in different classrooms are similar (similar ventilation rates per person). Natural ventilation was suggested by Lee and Chang [42] to be efficient in increasing the ventilation rates of classrooms. As offices are extremely large hubs in the network, reducing infection risks in offices will delay the peak infection. Hence, increasing ventilation rates in homes is essential to protect the public from infection from airborne diseases, whereas applying targeted control methods such as increasing ventilation rates to large location hubs may delay the rise of the epi-curve and provide time for the public to prepare for the outbreak.

### Advantages and limitations of our approaches

The advantage of our indoor contact network model is that only a small amount of data is required to build a model of the indoor contact network of a large city and to simultaneously capture the heterogeneous population contact pattern and uncertainty of the network structure. The indoor contact network in Hong Kong has a larger number of vertices with a higher degree of contacts than the people-people contact network in the city of Portland [18]. The highly connected graph developed in this study is potentially related to the common habits of eating out and using public transportation among inhabitants of Hong Kong.

Our limited input data (i.e. number locations, location visiting frequencies) and our assumptions (i.e. number of favorite locations), may influence the accuracy of our network topology. Further studies can address this problem by using more detailed data or fitting the network in a small-scale, real contact network investigation. This study also did not consider the geographical contact distribution, which may have a significant influence on the dynamics of infectious disease transmission [43, 44]; the local outbreaks cannot be predicted in the current model. Future studies are necessary to evaluate the geographical aspects of the social contact network and the role of possible transmission hubs that are not included in this study such as hospitals.

Contacts between friends are not simulated in our contact network model due to the difficulty of identifying the number of friends an individual may have within and outside the individual’s subgroup and the lack of social investigation data. The gathering activities are also hard to define in terms of frequency and location. Similarly, the individual diversity is not considered. Age is not integrated into the indoor contact network model due to the lack of data on age distribution within each population subgroup. In addition, the individual vaccine histories and the presence of super infectors have important effects on predicting the outbreak. A more complex model can be built when more detailed data is available.

Although only a limited number of intervention methods were compared, as increasing ventilation rate is the major concern of in this study, the effects of other intervention methods such as school closure, contact tracing, ultraviolet germicidal irradiation, and wearing masks could be evaluated in the same social contact network.

One significant limitation of this study is that we assumed that the disease (similar with smallpox) is transmitted only via the airborne route. In reality, respiratory infections may have multiple possible transmission routes [45, 46]. The transmission dynamics and the control effects of different methods might shift with the dominant transmission route or routes. However, the aim of the study is to demonstrate the effectiveness of ventilation in combination with other intervention methods in controlling a possible airborne disease outbreak on a social contact network, as well as our ability to simulate different scenarios on the theoretical basis. In another study by the authors [47], the role of ventilation in controlling the partially airborne influenza was investigated; it was found that increasing the ventilation rate significantly impacts on the transmission dynamics, even when the airborne route only contributes 20% to the total infection risk. Besides, as the methodology of constructing an indoor contact network provided by this study contains information about both individual-based social contact behavior and local environment transmission risks, a similar methodology could be adopted to model the multiple-routes-transmitted respiratory infections in large cities.

When applying the Wells-Riley model to estimate the local transmission rate, the selection of quanta generation rate is still a crucial problem. Most studies have applied a quanta generation rate calculated from historical outbreaks [10, 25, 28, 48, 49]. This method might lead to an over-estimation of the infectivity of the diseases due to the super-spreader effect [33]. Furthermore, the number of outbreak cases with sufficient information to estimate the quanta generation rate is very small, and a significant bias will be introduced by this small number of statistical data. However, other infectious diseases outbreak parameters, such as the basic reproductive number, are well documented for the past infectious disease outbreaks. In this study, we provide a method for investigating the correlations between basic reproductive number and quanta generation rate. The quanta generation in the previous outbreak can be estimated from the basic reproductive number by rebuilding the social contact network during the outbreak period.

## Supporting Information

S1 File Supplementary information. The following information is described in detail, e.g. construction of an indoor contact network, ventilation rate profile of indoor environments in Hong Kong, and determining the quanta generation rate.
(PDF)
