# Supplementary material for: Building Ventilation as an Effective Disease Intervention Strategy in a Dense Indoor Contact Network in an Ideal City
Source: PLoS One. 2016 Sep 9;11(9):e0162481. doi: 10.1371/journal.pone.0162481 (PMC5017609; doi:10.1371/journal.pone.0162481)
Supplement: S1 File — The following information is described in detail, e.g. construction of an indoor contact network, ventilation rate profile of indoor environments in Hong Kong, and determining the quanta generation rate. (PDF) [file pone.0162481.s001.pdf]

Supplementary Information for

Building ventilation as an effective disease intervention strategy in a dense indoor contact network in an ideal city

Xiaolei Gao, Jianjian Wei, Hao Lei, Pengcheng Xu, Benjamin J. Cowling, Yuguo Li

1. Construction of an indoor contact network

An actual indoor contact network can be affected by many factors such as the age, gender, occupation, personal habits, social status, geographical location, while many of these factors may correlate with each other. Hence, it is impossible to mathematically describe, accurately and completely, a real social contact network. To create a representative synthetic contact network and to simulate a possible infectious disease outbreak based on the network, we adopt a few simplifications.

1.1 Compartmentalization of population and indoor environments.

We separated population and indoor environments into representative groups and assumed that individuals in the same group share similar location visiting behaviors, and all of the indoor environments in the same group share similar characteristics such as the number of occupants, occupant density, and ventilation rate. Internal variations within each group can be simulated by assuming the possible distributions for different parameters.

Visiting time The complex patterns of individuals arriving at and leaving a location are not considered. We simplified the scenario by dividing each indoor environment into different sub-visiting locations that represent different time durations for visiting each indoor environment. Thus, several locations may represent the same indoor environment at a different time. Therefore this approach differentiates individuals who visit the same indoor environment at different times. We also assumed that individuals who choose to visit a location (an indoor environment for a fixed duration) come at the same time and leave at the same time. Hence, the visiting time of all possible visitors to a given location is the same.

Schedule We assumed that all of the individuals in a population group have the same location visiting schedule on weekdays. During a normal weekday, an individual may visit several locations in different location groups with a fixed order. For example, an individual may leave home at 8:00 am to go to office by bus; he arrives at his office at 9:00 am and stays there until 1:00 pm; he enters a restaurant for lunch at 1:00 pm; he goes back to his office at 2:00 pm and stays there until 6:00 pm; he has his dinner at a restaurant and then goes shopping; at 10:00 pm he takes the bus home. In real life, individuals may choose to go shopping during their lunch period. This variation is not simulated, as the incubation period of the disease takes a day or more, and thus the location visiting order may not influence the disease transmission dynamic.

Favorite locations To simulate the variations in an individual's daily choices, we assumed that for each step of an individual's schedule, he or she has a collection of favorite locations. Random contact is not considered in this model.

Visiting probability Visiting possibilities to an individual's favorite location will only be influenced by that individual's other possible choices and not by other individuals' choices.

Below we illustrate the detailed implementation methods for building an indoor contact network.

1.2 Compartmentalization of population and location groups

Age group-based social contact patterns have been adopted in many previous studies that used social contact data to simulate infectious disease outbreaks (Edmunds et al., 1997; Wallinga et al., 2006; Mossong et al., 2008). However, the directly measured social contact data in these studies largely depends on participants' recall. The reported contacts are limited to conversational contacts or face-to-face contacts. Hence, these data cannot be used to simulate the outbreak of airborne transmitted diseases, as exposure from a source patient is defined as sharing an indoor environment rather than direct contact (Edmunds et al., 1997). In this study, we used statistical data to simulate the location visiting behavior of individuals who have similar social activities.

There are many existing methods for classifying individuals according to similar social activities, such as age, gender, occupation, or personality. As illustrated in the main body of this study, many authors have argued that occupation has a dominant influence on individuals' social behavior. Therefore, we defined social groups according to representative occupations. Although statistical data describing the relative proportion of different occupations or economic activities are available from the Hong Kong Government, the divisions are rather rough, obscure, and overlap with each other. We preliminarily define eight social groups by combining the Hong Kong Government data on population groups with potential similar location visiting behavior profiles are summarized in Table A1.

Table A1 Sub-groups of individuals

Abbr.	Name	Description	Number
PH	Home stay	Individuals staying at home (housewives, retired people, young children, domestic helpers, etc.)	2,286,125[a]
PO	Office workers	Individuals working in offices or factories during the daytime	1,503,830[b]
PC	Classroom attendances	Students from kindergarten to university and teachers	1,267,850[c]
PR	Food service workers	Individuals working in restaurants	196,940[d]
PS	Sellers	Individuals working in any kind of shop	352,000[e]
PT	Drivers	Individuals driving or serving public transportation vehicles	10,375[f]
PP	Public workers	Individuals working in public places	64,980[g]
POT	Others	Individuals in the labor force without classification	1,175,000[h]
	Overall	Total population	6,857,100[i]

[a] Calculated with the non-labor force population data for 2006 (collected from the Census and Statistics Department's website http://www.censtatd.gov.hk/hong_kong_statistics/statistical_tables/index.jsp?charsetID=1&tableID=008, accessed July 2010); the dataset excludes a number of students enrolled in the 2007/2008 academic year (collected from the Hong Kong Government Education Bureau's website (http://www.edb.gov.hk/index.aspx?nodeID=92&langno=1, accessed: Jul. 2010) and a number of domestic helpers (Ho, 2004).
[b] Calculated from the government labor engagement data including all of the manufacturing, banking, financing, and other business industries (collected from the Hong Kong Government Census and Statistics Department's

website
http://www.censtatd.gov.hk/products_and_services/products/individual_statistical_tables/labour/index_cd_D525000707E_dt_detail.jsp accessed: Jun. 2010) and the number of current civil service employees (collected from the Hong Kong Government's website http://www.gov.hk/en/about/govdirectory/govstructure.htm accessed : Jun. 2010).

[c] Adopted from the government-released data on the number of students and teachers in 2007/2008 (collected from the Education Bureau's website http://www.edb.gov.hk/index.aspx?nodeID=92&langno=1 accessed: Jun. 2010), which excludes vocational training students.

[d] Adopted from the government-released data on people engaged in the restaurant industry (Census and Statistics Department HKGOV, 2007).

[e] Sum of the number of people employed in wholesale, retail, repair, and laundry services (Census and Statistics Department HKGOV, 2007).

[f] We assume that each public bus only has one bus driver. The number of public buses was collected from the website of the Transportation Department
(http://www.td.gov.hk/transport_in_hong_kong/public_transport/index.htm accessed: May 2010)

[g] Sum of the number of people employed in libraries, museums, cultural services, miscellaneous amusement services, recreational services, motion pictures, and other entertainment services (Census and Statistics Department HKGOV, 2007).

[h] Calculated from the rest of the data.

[i] Adopted from the government-released data for 2006 (Census and Statistics Department HKGOV, 2007)

Individuals not in the labor market, such as housewives, domestic helpers, and retired people, have different location visiting behavior than other individuals, as they do not have specific work or study locations with a potentially fixed number of daily contacts and their frequency of shopping might be much higher than other groups. Hence, we classify them in one group as home staying individuals and name the group **PH**.

Individuals working in locations such as libraries, museums, theatres, and other public places may engage with a relatively large number of random contacts in large spaces. As the size of a location is an important factor in the ventilation rate and the probability of airborne infection, we classified these individuals into a separate group called public workers (**PP)**. Some other occupations such as construction, delivery, and outdoor promotion do not have a significant number of work-related indoor contacts, hence they were grouped together as others (**POT**).

The location categories are listed in Table A2. To reduce the simulation cost, we used one day as the outbreak simulation time step and assume that individuals' infection statuses will only be altered daily. However, the unit of time in individuals' location visiting schedules might be an hour or even minutes. To coordinate the two different units, we assumed that individuals in the same location group have the same daily schedule; therefore, the possible locations that an individual visits during a day are organized into a sequence. When this assumption is made, the time of a visit to a location such as a restaurant has to be specified, because individuals may choose to visit these locations at different times, and the assumption that all of them visit a location together will cause an overestimation of social contacts. Hence, we divided each location in the restaurants, shops, public places, and transportation location groups into several locations, each representing different time periods that individuals may spend at these locations.

As the probability of eating out for lunch may be different than the probability of eating out for dinner, we also divided location group LR into sub-groups LRL (restaurants during lunch time) and LRD (restaurants during dinner time). Each bus or train travels the same route back and forth many times during a day. However, to simplify the model we only considered transportation during rush hours, hence LT is divided into LTM (transportation in the morning) and LTA (transportation in the afternoon).

The number of locations in location group LH (homes) can be replaced by the total number of households if we assume that one home is equal to one indoor environment. However, the number of offices in Hong Kong is hard to estimate. The only available data are the total floor area of private offices in Hong Kong. Although the area of an average office can be estimated from other studies and used to calculate the number of offices, without the data for government offices the estimate is inaccurate. Hence, we estimated the number of offices using the size of the population group PO together with the data on occupant density from the EPD study (EPD 1995).

The number of classes in schools can be calculated from government data available on the Education Bureau's website (http://www.edb.gov.hk/index.aspx? nodeID=92 &langno =1 accessed : Jan. 2010) and this can be combined with the overall number of students and average class sizes at different education levels (see Table A 3). Due to the lack of data on the average post-secondary class size, we assume it to be 40 students per a class. The data for other location groups were drawn from government websites and are listed in Table A2.

Table A2 Sub-groups of locations

Abbr.	Name	Description	No. of indoor environments	No. of locations
**LH**	Home	Homes, including anywhere that individuals stay at night, including university dormitories.	220,100[a]	2,201,000
**LO**	Office	Offices where members of sub-group PO work during the day time.	30,000[b]	30,000
**LC**	Classroom	Classrooms of kindergarten, primary schools, high schools, universities, and training centers.	38,807[c]	38,807
**LRL**	Restaurant	Restaurants where individuals have lunch (one restaurant can be divided into four locations according to visiting time 12:00-12:30, 12:30-13:00, 13:00-13:30 , 13:30-14:00).	18,000[d]	72,000
**LRS**	Restaurant	Restaurants where individuals have supper (one restaurant can be divided into four locations according to visiting time 18:00-18:30, 18:30-19:00, 19:00-19:30 , 19:30-20:00).	18,000	72,000
**LS**	Shop	All kinds of shops including shopping malls, supermarkets, retail shops, etc. (one shop can be divided into four locations according to visiting time of 12:00-14:00, 16:00-18:00, 18:00-20:00 or 20:00-22:00).	87,300[d]	349,200
**LTM**	Transportation	Buses, MTR, and KCR that people take in the morning.[f]	-	70,134[e]
**LTA**	Transportation	Buses, MTR, and KCR that people take in the afternoon.[f]	-	70,134[e]
**LP**	Public location	Public places, museums, cinemas, gyms, karaokes (one place can be divided into four locations according to visiting time of 12:00-14:00, 16:00-18:00, 18:00-20:00 or 20:00-22:00).	4,940[d]	19,760

[a] Adopted from the data published on the website of the Hong Kong Housing Authority of the Hong Kong Government (http://www.housingauthority.gov.hk/en/aboutus/resources/graphicguides/0,,1-0-2755--0,00.html accessed May 2010).
[b] Estimated according to the average occupants value given in the EPD indoor environment study (EPD 1995) and the number of office workers in Table A1
[c] See Table A3.

[d] Adopted from the largest restaurant search engine (http://www.openrice.com/page/about.htm accessed: Aug. 2009)
[e] Calculated by summarizing the number of public buses as given on the Hong Kong Government Transportation Department's website (http://www.td.gov.hk/transport_in_hong_kong/public_transport/index.htm accessed: Feb. 2010) and the number of MTR spaces (locations) estimated according to the number of weekday passengers, using the assumption that each MTR location contains 80 passengers.
[f] Each trip is defined as a location; private cars are not included in the location group.

Table A3 Class sizes at different education levels

Education level	No. of students	No. of teachers	No. of class attendants[a]	Average class size	Average class attendants[b]	No. of classes
**Kindergarten**	140,800[c]	10,355[c]	151,155	20.3[c]	21.3	6,936
**Primary**	410,500[c]	22,787[c]	433,287	31.7[c]	32.7	12,950
**Secondary S1-S5**	418,678[c]	29,106[c]	447,784	37.8[c]	38.8	11,076
**Secondary S6-S7**	637,27[c]		63,727	30.3[c]	31.3	2,103
**Higher**	150,200[c]	19,902[c]	170,102	40[d]	41	5,742
**Overall**	1,183,905	82,150	1,266,055	NA	NA	38,807

[a] Number of students plus number of teachers.
[b] One teacher is included
[c] Collected from the Education Bureau's website (http://www.edb.gov.hk/index.aspx?nodeID=92&langno=1, accessed: May 2010)
[d] Assumed to be higher than the number of students in other education levels.

1.3 Building community of population and locations

Neglecting households larger than seven people, we plot the distribution of household size in Figure A1. (The data are drawn from the Hong Kong Government Census and Statistics Department's website http://www.censtatd.gov.hk/hong_kong_statistics/statistical_tables/index.jsp?charsetID=1&tableID=161 accessed: May 2010). The correlation between occupation and household size was neglected in this study due to the limited available data. Due to the lack of data for private housing in Hong Kong, the average living space (12.5 m^2/person) in public rental housing 2009 (collected from the website of the Hong Kong Housing Authority http://www.housingauthority.gov.hk/en/aboutus/resources/graphicguides/0,,1-1727-2755,00.html accessed : Jan. 2010) was adopted as the average living space in Hong Kong. Other studies have shown that the average occupancy density may be between 10 to 15 m^2/person (Lee et al., 1999; Chao, 2001; Wan and Yik, 2004).

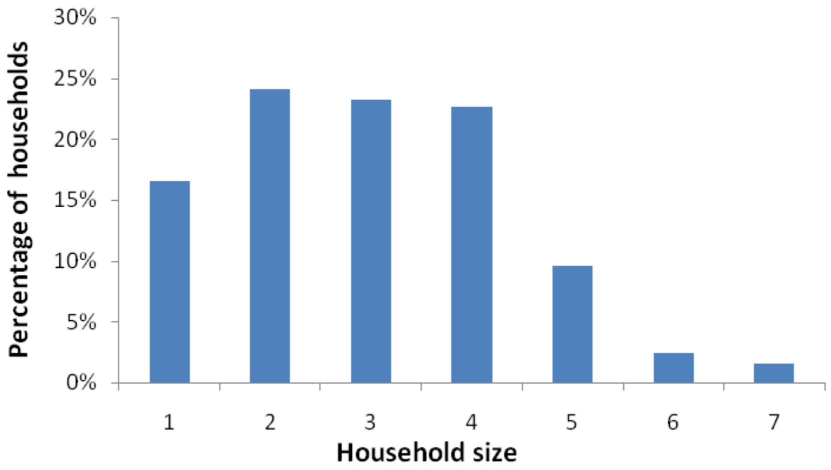

Figure A1 Distribution of domestic household size, assuming a maximum of seven household members

The data used to calculate the number of occupants in office locations is drawn from a consultancy study of 40 offices conducted by the Hong Kong Environmental Protection Department (EPD) (EPD, 1995). The best fit for this data is a log-normal distribution (using the maximum likelihood method), as shown in Figure A2 (a). The correlation between the number of occupants and occupant density is not significant according to the EPD study. Hence, we assume that they are independent. The best fit for the office occupant density data (EPD, 1995) is also the lognormal distribution, as shown in Figure A2 (b).

The number of classroom attendants in Hong Kong may be more consistent than the number of occupants in other types of locations. Hence the distribution of class size was fitted from the average number of class attendants at different education levels (listed in Table A3) using the maximum likelihood method. The best fit is log-normal (3.46, 0.23).

In contrast, other locations such as restaurants and shops, do not have a fixed number of daily visitors. As the number of occupants in location groups LO and LC both best fit a log-normal distribution, we assumed that other location groups can also be fitted to this profile, which is suitable when there are a few locations that are significantly larger than the others. The mean-log and SD-log of the distributions of other location groups are estimated based on the total number of locations and the average number of occupation-related visitors in these locations. Please note that the occupant density distributions in location groups LS and LP are estimated based on a small number of studies (see Table A4).

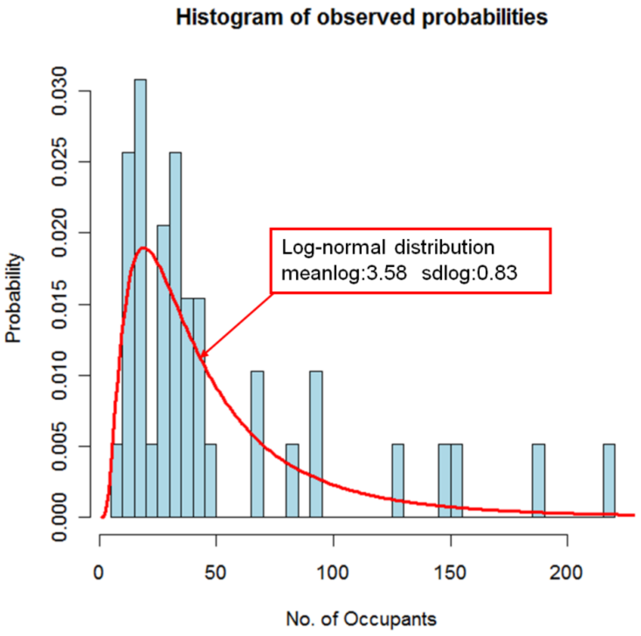

(a)

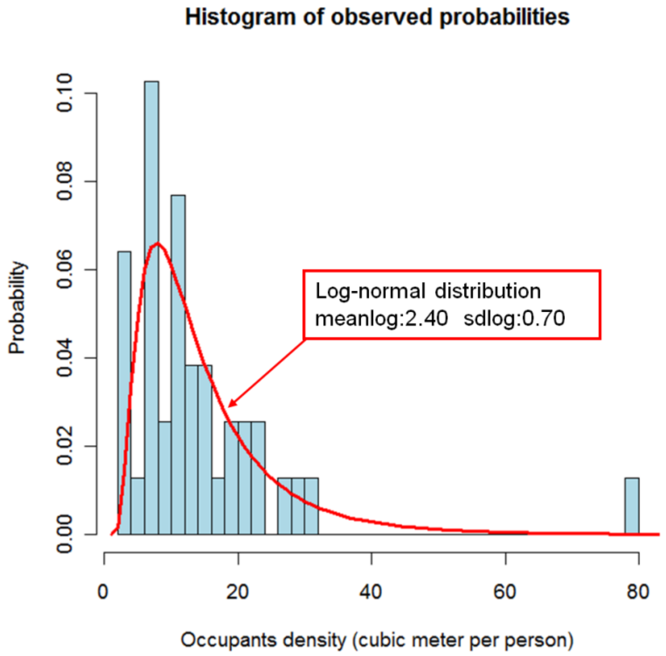

(b)

Figure A2 (a) The number of occupants of offices (EPD, 1995), and best fits (log-normal distribution). (b) Occupants density data (EPD, 1995) and best fits (log-normal distribution).

Table A4. Size distribution of location groups

Abbr.	No. of occupants	Occupant density (m^3/person) [a]	Minimum size [b]	Maximum size [b]
**LH**	See Table A1	Constant: 31.2 [d]	1	7

**LO**	Log-normal (3.58, 0.83) [e]	Log-normal (2.4, 0.7) [e]	1	200
**LC**	Log-normal (3.46 , 0.23) [f]	Log-normal (1.87, 0.21) [g]	10	60
**LR(LRL,LRS)[c]**	Log-normal (2.00, 0.90)[h]	Log-normal (4.2, 0.1) [i]	1	100
**LS[c]**	Log-normal (1.00, 0.90)[h]	Log-normal (4.1, 0.1) [j]	1	100
**LT(LTM,LTA)[c]**	0 or 1	20 ,40 or 80 [k]	0	1
**LP[c]**	Log-normal (2.20, 0.90) [h]	Log-normal (5.3, 0.1) [j]	1	100

[a] Estimated value
[b] Estimated value
[c] Occupants is defined as occupation-related individuals working in these locations.
[d] Calculated from the average living space, $12.5m^2$/ person, and room height, 2.5 m (Lee et al., 2002b) .
[e] Fitted from the number of occupants data drawn from the EPD study (EPD, 1995).
[f] Fitted from average class sizes for different education levels (Census and Statistics Department HKGOV, 2006) together with the number of students in each level in 2007/2008 (Education Bureau website (http://www.edb.gov.hk/index.aspx?nodeID=92&langno=1).
[g] Estimated from Lee and Chang (2000) and Lee et al. (2002a).
[h] Assuming that the occupants number follows a log-normal distribution. The values of the log-mean and log-SD are roughly estimated based on the average number of occupants (service workers in restaurants: 16.5/restaurant, sellers in shops: 4 person/shop, workers in public places: 13.15 person/public places) and calculated according to Table A1 and Table A2 due to the limited data.
[i] Estimated from Lee et al. (2001)
[j] Assumed without any available data.
[k] Estimated from the average number of passengers in a mini-bus (25%), single-decker bus (0.5%), double-decker bus (32%), and MTR (41%) and the government website data on the number of different vehicles and their daily capacity (http://www.gov.hk/en/residents/transport/publictransport/index.htm) .

1.4 Estimation of location-visiting matrix

To describe the indoor-visiting behavior of the population in each different group, a proportion matrix, *M*, is used to represent the different proportions of individuals in all of the population groups who will visit different types of locations.

$$M = \begin{bmatrix} M_{PH,LH} & \cdots & M_{PH,LT} \\ \vdots & \ddots & \vdots \\ M_{POT,LH} & \cdots & M_{POT,LT} \end{bmatrix} \tag{A1}$$

where $M_{PH,LH}$ represents the proportion of individuals in population group PH who might visit a location in location group LH during a simulation day. To capture the variation within population groups, we assumed that the probabilities that an individual in any population group visiting a location in any location group follow a normal distribution. Hence, $M_{PH,LH}$ represents the mean probability that individuals in group PH visiting a location in group LP. Due to the lack of relevant data, the standard deviation is assumed to be 10% of the mean probability for all population groups.

The estimated of the number of daily visitors to different location groups are first calculated from the government household expenditure data and are listed in Table A5. The data for all visitors, together with other government investigations, are used to estimate the location-visiting proportion matrix *M*. As suggested by Edmunds et al. (1997), the contact patterns on weekdays and weekends are significantly different in terms of number of contacts and contact locations. Given the differences in social behavior on weekdays and weekends, we also divide *M* into a weekdays proportion matrix, M_d, given in Table A6, and a weekends proportion matrix, M_e , given in Table A7.

The visiting probabilities of LRL and LRR on weekdays were estimated according to the lunch eating-out data in 2007 published by the Department of Health of the Hong Kong Government (http://www.chp.gov.hk/en/behavioural/10/280.html). The

government data shows that there is significant variation in the eating-out habits of different occupations. Hence, the eating-out data are adjusted with the labor force ratio data.

As the eating-out habits of students are not given in the Department of Health's study, we assumed that students at the secondary level or higher have the same eating-out habits as adults, whereas younger students will only eat at home. The probability of eating out for students (population group PC) can then be calculated given the ratio of students at different education levels (http://www.edb.gov.hk/index.aspx?nodeID=92&langno=1). The overall estimated number of visitors to locations LRL and LRR based on the location visiting probabilities is consistent with the number of visitors estimated from the household daily expenditure survey (see Table A5).

The data on the main mode of transportation of students and the working population were adopted from the 2006 population by-census (Census and Statistics Department HKGOV, 2006); these data were used to estimate the proabilities of group PO, PC, PR, PS, PP, and POT visiting LTM and LTA. Here, we assumed that individuals who work or attend classes have the same possibility of visiting LTM and LTA, hence the visiting possibility of population group PH to these locations can be calculated from the total number of visitors to LTM and LTA. This was estimated from the household daily expenditure data by excluding the data for other groups.

Table A5 Estimation of number of visitors

Sub-groups	Monthly spending per family (HKD) [a]	Overall spending in Hong Kong per day [b](HKD)	Estimated spending per each visit (HKD)	Number of visitors per day
LRL+LRS	3,078	225,822,600	30	7,527,420
LS	4,402	322,960,067	100	3,229,601
LTM+LTA	950	69,698,333	7	9,956,904
LP	70	5,135,666	40	128,391

[a] Adopted from the Household Expenditure Survey on the Census and Statics Department's website (http://www.censtatd.gov.hk/products_and_services/products/publications/statistical_report/social_data/index_cd_B1130214_dt_detail.jsp)
[b] We assume that there are three people in a household.

Table A6 Weekday location-visiting proportion matrix, Md, of population groups vs. location groups

Groups	PH	PO	PC	PR	PS	PT	PP	POT
LH	100.0%	100.0%	100.0%	100.0%	100.0%	100.0%	100.0%	100.0%
LO	0	100.0%	0	0	0	0	0	0
LC	0	0	100.0%	0	0	0	0	0
LRL	43.0% [a]	74.8% [a]	57.0% [c]	74.8% [b]	74.8% [a]	74.8% [a]	74.8% [a]	74.8% [a]
LRS	22.7% [a]	38.3% [a]	29.8% [c]	85.7% [a]	38.3% [a]	38.3% [a]	38.3% [a]	38.3% [a]
LS	69.4% [d]	34.7% [d]	34.7% [d]	34.7% [d]	85.7% [b]	34.7% [d]	34.7% [d]	34.7% [d]
LTM	36.3% [g]	78.4% [e]	70.0% [f]	78.4% [e]	78.4% [e]	85.7% [e]	78.4% [e]	78.4% [g]
LTA	36.3% [g]	78.4% [e]	70.0% [f]	78.4% [e]	78.4% [e]	85.7% [b]	78.4% [e]	78.4% [e]
LP	36.0% [h]	9.0% [h]	9.0% [h]	9.0% [h]	9.0% [h]	9.0% [h]	85.7% [h]	9.0% [h]

[a] Adjusted data from the investigation of lunch eating out habits published by the Department of Health of the Hong Kong Government (http://www.chp.gov.hk/en/behavioural/10/280.html)
[b] Calculated based on a 6-day work week; for this group, the possibility of working on the weekdays is the same as on weekends.
[c] As the government's investigation of eating out habits did not cover people under 18, we assume that kindergarten and primary school students do not eat out for lunch and that older students have the same habits as

working adults. The proportion of kindergarten and primary school students is taken from the Education Department's website.
[d] We assume that the shop visiting rate of people in group PH is twice as high as the visiting rate of other groups. The estimates are calculated based on Table A5.
[e] Adopted from the government's transport to work data (Census and Statistics Department HKGOV, 2006).
[f] Adopted from the government's transport to study data (Census and Statistics Department HKGOV, 2006).
[g] Calculated from the total number of visitors in Table A5 and the visiting possibility of other groups.
[h] Estimated according to the number of visitors in Table A5 by assuming that the visiting possibility of group PH on weekdays and all of the groups on weekends to location group LP is four times higher than that of all groups except PH on weekdays.

Table A7 Weekend location-visiting proportion matrix, M_e, of population groups vs. location groups

**Groups**	**PH**	**PO**	**PC**	**PR**	**PS**	**PT**	**PP**	**POT**
**LH**	100.0%	100.0%	100.0%	100.0%	100.0%	100.0%	100.0%	100.0%
**LO**	0	0	0	0	0	0	0	0
**LC**	0	0	0	0	0	0	0	0
**LRL**	43.0%[a]	43.0%[a]	43.0%[a]	85.7%[b]	43.0%[a]	43.0%[a]	43.0%[a]	43.0%[a]
**LRS**	44.0%[a]	44.0%[a]	44.0%[a]	85.7%[b]	44.0%[a]	44.0%[a]	44.0%[a]	44.0%[a]
**LS**	69.4%[c]	69.4%[c]	69.4%[c]	69.4%[c]	85.7%[b]	69.4%[c]	69.4%[c]	69.4%[c]
**LTM**	36.3%[d]	36.3%[d]	36.3%[d]	36.3%[d]	36.3%[d]	85.7%[b]	36.3%[d]	36.3%[d]
**LTA**	36.3%[d]	36.3%[d]	36.3%[d]	36.3%[d]	36.3%[d]	85.7%[b]	36.3%[d]	36.3%[d]
**LP**	36.0%[e]	36.0%[e]	36.0%[e]	36.0%[e]	36.0%[e]	36.0%[e]	85.7%[b]	36.0%[e]

[a] We assume that the possibility of eating out for lunch and dinner for all groups on weekends are the same as eating out for dinner on weekdays.
[b] Calculated based on a 6-day work week; for this group, the possibility of working on weekdays is the same as on weekends.
[c] We assume that the shop visiting rate of people in group PH is twice as high as the visiting rate of other groups. The estimates are calculated based on Table A 5.
[d] We assume this is the same as the visiting possibility of group PH on weekdays.
[e] Estimated according to the number of visitors in Table A5 by assuming that the visiting possibility of group PH on weekdays and all of the groups on weekends to location group LP is four times higher than that of all groups except PH on weekdays.

1.5 Construction of the people-location visiting network

In this study we assumed that the location-visiting probabilities of the individuals in each population group for different location types follow a normal distribution and with a mean equals to the location-visiting proportion and a standard deviation of 10% of the mean value. Hence, a social indoor contact network can be built by connecting individuals with the locations that they may visit.

However, connecting every individual with every location would have an extremely high computational cost. To reduce the size of the interaction matrix of individuals and locations, we assumed that individuals only choose locations from a collection of their favorite locations and that all individuals in each population group have the same number of favorite locations in each of the different location groups. The assumed number of favorite locations for each group and location are listed in Table A8. The time use patterns of each population group were assumed to be identical. The visiting time of each population group to different location groups was estimated according to government social statistical data; the estimates are given in Table A9.

Table A8 Number of favorite locations

**Groups**	**PH**	**PO**	**PC**	**PR**	**PS**	**PT**	**PP**	**POT**
**LH**	1	1	1	1	1	1	1	1
**LO**	0	1	0	0	0	0	0	0
**LC**	0	0	1	0	0	0	0	0
**LRL**	4	4	4	4 [a]	4	4	4	4

**LRS**	4	4	4	4[a]	4	4	4	4
**LS**	4	4	4	4	4[a]	4	4	4
**LTM**	4	4	4	4	4[a]	4	4	4
**LTA**	4	4	4	4	4	4	4[a]	4
**LP**	4	4	4	4	4	4	4[a]	4

[a] Different locations divided by different visiting periods in the same location.

Table A9 Estimation of visiting times on weekdays (hours per location)

**Groups**	**RH**	**PO**	**PC**	**PR**	**PS**	**PT**	**PP**	**POT**
**LH**	14.1[a]	11.6[a]	11.3[a]	11.6[a]	11.6[a]	11.6[a]	11.6[a]	11.6[a]
**LO**	0	8.4[a]	0	0	0	0	0	0
**LC**	0	0	7.8[a]	0	0	0	0	0
**LR**	0.75	0.75	0.75	1.25[b]	0.75	0.75	0.75	0.75
**LS**	1.0	1.0	1.0	1.0	2.1[c]	1.0	1.0	1.0
**LT**	0.5	0.5	0.5	0.5	0.5	1.25[b]	0.5	0.5
**LP**	2.0	2.0	2.0	2.0	2.0	2.0	8.4	2.0

[a] Estimated according to the time use pattern data available on the Census and Statistics Department's website.(http://www.censtatd.gov.hk/products_and_services/products/publications/statistical_report/social_data/index_cd_B1130214_dt_detail.jsp).
[b] One location with eight different periods is used for individuals who work in one location; the exposure time in each location is calculated using average work hours.

In reality, an individual's choice of location might be influenced by behaviors of others who arrived at the same location earlier and may change over time. However, as a probability model is applied in this study, the effect of priority is neglected.

As the probabilities of individuals choosing their favorite locations are independent, which means that the selection of favorite locations of an individual cannot be limited by other people's choices. Hence, individuals should not queue to be linked to their favorite locations and their choices should be random. However, all of the locations have size limitations. To address this issue, we first randomly connected individuals with their favorite locations; a recursion algorithm was then adopted to randomly select individuals in overflowed locations and rearrange their choices. The flow chart of the recursion algorithm is given in Figure A3.

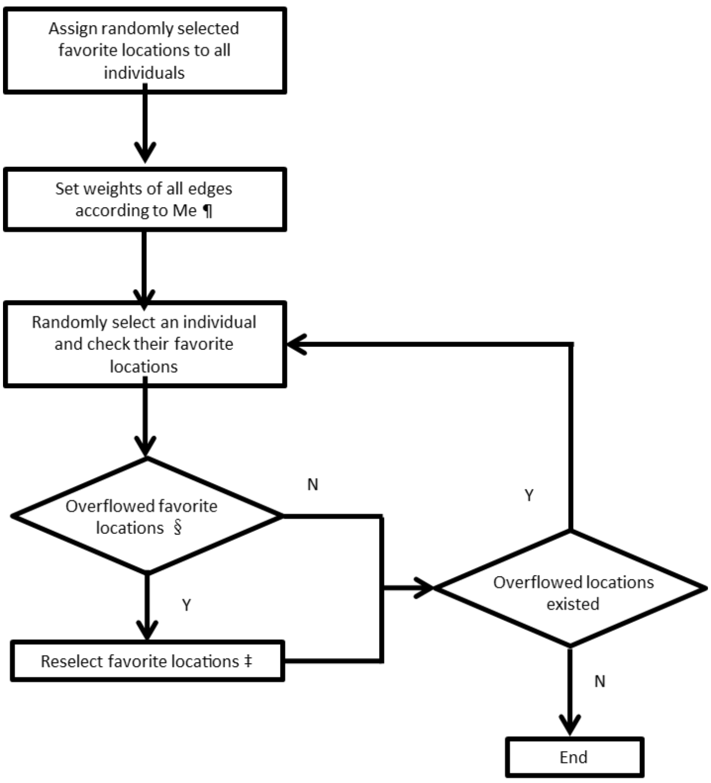

Figure A3 Recursion algorithm for connecting individuals with their favorite locations. If there is more than one location selected in the same location group, the visiting probability is first randomly distributed to these locations. ¶Weights are then readjusted according to the size of these locations, under the assumption that larger locations will be visited with higher possibility. §Overflowed here is defined as the expectation that the number of visitors exceeds the capacity of the location. ‡ When one favorite location is overflowed and needs to be re-selected, the visiting probabilities of the other favorite locations in the same location group, as selected by the individual, need to be generated again due to the changed location choices.

After constructing all of the edges with no overflowed locations, the location visting matrix is then a data file that is used in the SEIR model.

2 Ventilation rate profile of indoor environments in Hong Kong

2.1 Search strategies

During the past several decades, indoor air quality has been the subject of wide public concern due to its effects on health, including its role in pollutant exposure and infectious diseases transmission. Previous studies of ventilation rates (m^3/s) or air change rates (air change per hour, ACH), an important indicator of indoor air quality, have examined both its effects on health and its relationship with different indoor pollutants (Godish and Spengler, 1996; Seppanen et al., 1999; Wargocki et al., 2000).

In this study, ventilation is a crucial governing parameter for calculating the transmission probability of an airborne disease. Although ventilation rate data can be acquired from different standards, the difference between actual ventilation rates and theoretical ventilation rates cause estimation bias in the results. Moreover, household ventilation rates are hard to estimate due to the influence of weather, building design,

location, and occupants' habits. Hence, there is a need to establish the distribution of ventilation rates in different types of buildings.

To construct a basic ventilation scenario for Hong Kong, this study first reviewed the papers that provided ventilation data for the location sub-categories, including households, offices, schools, restaurants, shops, libraries, and other public places, that are available in the following databases:

ISI Web of Science, (1900-present);
ScienceDirect (1823-present);
PubMed (1865-present);
Engineering village 2 (1969-present); and
Google Scholar, http://scholar.google.com.hk/.

The search strategy was as follows. The first four databases were thoroughly searched; Google Scholar was only used as an additional source to search for full papers. Government studies from the Hong Kong government's website (http://www.gov.hk/) were also used.

The keywords, listed in Table A 10, were separated into three groups: **Group 1** words were related to ventilation and ventilation rate, **Group 2** words were related to sub-group building names; and **Group 3** contained only the location of the study. In the searches, the keywords in **Group 1** and **Group 2** were connected with OR, and the keywords in **Group 1**, **Group 2,** and **Group 3** were connected with AND.

The search strategy and search results are given in Table A11. The ISI Web of Science yielded only 32 papers because this database does not provide a search field for abstracts. The search results were exported to an Endnote file and duplicated references were removed. Finally, 4,833 articles were found in these databases. One government study was also found on the EPD website. As the study of ventilation in vehicles might also be relevant to this study, an additional search was carried out for studies of ventilation in public transportation, with Group 3 words removed. Only complete papers written in English that measured ventilation rates were adopted. Among the studies in which ventilation rate or indoor air quality were measured by CO_2 concentration, only those that specified occupation density were selected.

Table A10 Keywords

Group name		Keywords
**Group 1**		ventilation rate, air change rate, air exchange rate, ACH,<br>air supply, supply air,<br>outdoor/outside air, fresh air, ventilation,<br>airflow or air flow,<br>indoor air, air quality
**Group 2**	**Homes:**	house, households, homes, dwellings, residence, residential, apartments
	**Offices:**	offices, commercial buildings
	**Schools:**	schools, classrooms, universities
	**Restaurants:**	restaurants, canteens, café, bars
	**Shops:**	shops, shopping malls, shopping centers
	**Libraries:**	libraries
	**Public transportation**	buses, vehicles, automobiles, trains, MTR, tramps
**Group 3**		Hong Kong

Table A11 Search strategy and results

Data bases	Search strategy	Number of articles
ISI Web of Science	Titles searched for keywords from Group 1 and Group 2 and addresses searched for Group 3 words	267
	Public transportation searched for, without Group 3 keywords	272
ScienceDirect	Titles, abstracts, and keywords searched for keywords from Group 1 and Group 2; the full texts searched for keywords from Group 3	388
	Public transportation searched for, without Group 3 keywords	1,248
PubMed	Titles and abstracts searched for keywords from Group 1 and Group 2; all fields searched for keywords from Group 3	1,462
	Public transportation searched for, without Group 3 keywords	22
Engineering village 2	Subjects, titles, and abstracts searched for keywords from Group 1 and Group 2; all fields searched for keywords from Group 3	512
	Public transportation searched for, without Group 3 keywords	1,467

The data on ventilation rates (l/s, l/s•person) or air change rates (ACH) in the selected articles were collected according to the above-mentioned categories. If only CO_2 concentration and number of occupants were given, we roughly estimated ventilation rate by assuming that the given CO_2 concentration reaches a steady-state, that all humans generate the same rate of CO_2, and that other indoor CO_2 sources are negligible.

The mass balance equation of CO_2 concentration can be expressed by the following equation

$$V\frac{\mathrm{d}C_{\mathrm{CO_2,indoor}}}{\mathrm{d}t} = q(C_{\mathrm{CO_2,indoor}} - C_{\mathrm{CO_2,outdoor}}) + G\,, \quad \text{(A2)}$$

where q is the ventilation rate (m^3/h) and G is the source generation rate of CO_2 (m^3/h). In the steady state, Equation (A2) becomes

$$q = \frac{G}{(C_{\mathrm{CO_2,indoor}} - C_{\mathrm{CO_2,outdoor}})}\,. \quad \text{(A3)}$$

The human respiratory CO_2 generation rate is assumed to be 0.01872 m^3/h (Cao et al., 1992). Hence the air change rate (ACH) in an indoor environment of volume V (m^3) with n occupants can be calculated as

$$\mathrm{ACH} = \frac{n \times 0.01872}{(C_{\mathrm{CO_2,indoor}} - C_{\mathrm{CO_2,outdoor}})V}\,. \quad \text{(A4)}$$

2.2 Results of the research on ventilation rates

Homes

The eight studies with adequate information about ventilation rates, and the eight studies with original data on ventilation rates are listed in Table A12. Given the uncertainty in the number of occupants in the average home and the indoor sources such as burning and cooking, we only used the ventilation rates measured by the tracer

gas test.

Table A12 Ventilation rates of homes tested in the studies

No.	No. of locations	Test method	Volume (m^3)	Ventilation type	ACH	Ref.
**1**	5	Tracer gas CO_2	92.2	Natural ventilation Windows opened	6.19	(Chao and Tung, 2001)
				Windows closed	1.52	
**2**	11	Tracer gas SF_6	-	Windows closed	0.36	(Chan et al., 2006)
				Air conditioner	0.65	
**3**	10	Tracer gas CO_2	142.7	Natural ventilation windows opened	5.42	(Chao, 2001)
**4**	10	Tracer gas CO_2	137	Natural ventilation	2.1	(Chao et al., 1997)
			160	Window-type air conditioner on	0.7	
**5**	5	CO2 monitors	12	Split-type air conditioner	0.5	(Lin and Deng, 2003)
				Window-type air conditioner	1.09	
**6**	18	Tracer gas CO_2	122.5	Windows closed	0.13	(Man and Yeung, 1999)
**7**	25	Tracer gas SF_6	18.36	Air conditioner off	0.38	(Lam et al., 2007)
				Air conditioner on	0.76	
**8**	1	CO_2 monitors	13.9	Windows closed air conditioner on	0.34	(Lee and Yu, 2000)
				Windows closed air conditioner off	0.24	
**9**	10	Tracer gas CO_2	12.18	Natural ventilation windows closed	0.49	(Chan and Ho, 2005)
**10**	35	Tracer gas	-	Windows ether opened or closed	4.66*	(Chao and Wong, 2002)

* Ventilation rate is always larger than 3.5 ACH with open windows and lower than 3.5 ACH with closed windows.

The results listed in Table A12 show that ventilation rates in households are largely influenced by the type of ventilation system. If all of the doors and windows are closed, the ventilation rate will be generally lower than 0.5 ACH, depending on the leakage of the building and the difference between the indoor and outdoor weather conditions. One study also showed that window-type air conditioners can achieve ventilation rates twice as large as those of split-type air conditioners. If an air conditioner is used with the windows and doors closed, the ventilation rate is slightly higher, about 0.8 ACH. When natural ventilation is adopted with all of the windows open, an average ventilation rate of 5 ACH can be achieved. However, the natural ventilation rates for different weather conditions and locations in Hong Kong are not available in the literature. A more comprehensive measurement study is needed to understand the ventilation profile of naturally ventilated homes in Hong Kong.

Offices

Three of the four field studies of ventilation rates in offices used the tracer gas test and one study provided the CO_2 concentration and number of occupants in tested indoor environments, as shown in Table A13. The collected ventilation rates, listed in Table A13, show that the ventilation rate in offices is around 1 to 2 ACH.

Table A13 Ventilation rates for offices tested in the studies

No.	Number of tested locations	Test method	Average room volume (m3)	ACH	Study

**11**	40	Tracer gas SF_6	193	0.88	(EPD, 1995)
**12**	10	Tracer gas SF_6	262	1.86	(Chao and Chan, 2001)
			5,000	1	
**13**	2	Tracer gas CO_2	1,400	2.96	(Law et al., 2001)
			112	1.15	

Table A14 Ventilation rates of offices estimated from CO_2 concentration

No.	Number of tested locations	Average room volume (m^3)	ACH	Study
**14**	10	160	2.53	(Lee et al., 2002a)

Schools

There are no studies that test the ventilation rate in schools using the tracer gas method. Two studies provide CO_2 concentration, as shown in Table A15. The ventilation rates are calculated using Equation (A4). According to the real time CO_2 concentration curve of one mechanically ventilated classroom, the highest CO_2 concentration can remain steady for a few hours, indicating that the peak concentration might be a steady state concentration. Hence, the use of an average CO_2 concentration might cause an overestimation of the ventilation rate.

Table A15 Ventilation rates of schools as estimated from CO_2 concentrations

No.	Number of locations	Average room volume (m3)	Number of occupants	Ventilation type	ACH	Study
**15**	5	201.2	41	Mechanical ventilation	3.4 (1.55)*	(Lee and Chang, 2000)
			39	Natural ventilation	16.7#	
**14**	10	214.2	32	Mechanical ventilation	5.08(2.06)^	(Lee et al., 2002a)

* Average CO_2 concentration is used in the estimation. If peak values are used, the ventilation rate is 1.55 ACH.
# Results from naturally ventilated classrooms with ceiling fans
^Average CO_2 concentration is used in the estimation. If peak value is used, the ventilation rate is 2.06 ACH.

The results show that naturally ventilated schools can achieve a much higher ventilation rate than other schools. However, the experiment was only for a single time period. Long-term average ventilation rates can be influenced by many factors such as weather conditions and might also change with the seasons. More studies are needed to measure the year-round ventilation rate in schools.

Shops

Only one study of shops uses a tracer gas test. Although many studies provide CO_2 concentrations in shops and shopping malls, occupancy density is hard to estimate in large shops. Hence, we only used the data from tracer gas tests (see Table A16).

Table A16 Ventilation rates for shops tested in the studies

No.	Number of tested locations	Test method	Average room volume (m3)	ACH	Study
12	1	Tracer gas SF_6	1650	0.9	(Chao and Chan, 2001)
			521	2.5*	

* Customer Services Centre here was ranged in the group of shops .

Libraries

The identified ventilation rate in libraries in Hong Kong, shown in Table A18, is similar to the ventilation rate in offices. However, the tested library rooms are small compared to office spaces. The ventilation rate in large volume locations is largely unknown.

Table A17 Ventilation rates for libraries tested in the studies

No.	Number of tested locations	Test method	Average room volume (m3)	ACH	Study
12	2	Tracer gas SF_6	374	1.4	(Chao and Chan, 2001)

Public transportation

Most of studies of ventilation in public transportation (Chan, 2003; Mui and Shek, 2005) use CO_2 as an indicator. However, the number of occupants in transportation locations changes rapidly, hence the ventilation rate cannot be estimated from the CO_2 level. There are no studies of public transportation in Hong Kong that use the tracer gas test to measure ventilation rates. Hence, studies from other places are used (see Table A 18).

Table A18 Ventilation rates for transportation locations tested in the studies

No.	Number of tested locations	Test method	Average room volume (m3)	ACH	Study
**13**	6	Tracer gas SF6	40	2.6-4.55	(Rim et al., 2008)

2.3 Summary

There are only 15 studies of 210 different locations in Hong Kong that provided adequate information about ventilation rates. Thirteen measure ventilation rates with the tracer gas test, and the ventilation rate can be estimated in the other two studies from the CO_2 concentrations.

Four studies provided ventilation rates for closed apartments with the air conditioner off. The studies suggest that ventilation rates are consistently below 0.5 ACH. Only two experiments in Study 1 found ventilation rates higher than 1 ACH; this high value is due to the higher floor level and leaky windows in these two apartments (Chao and Tung, 2001). As shown in Table A12, natural ventilation with open windows commonly gives ventilation rates above 3 ACH; the ventilation rate ranges from 0.3 to 1 ACH with air conditioners. Window-type air conditioners provide higher ventilation rates than split-type air conditioners (Lin and Deng, 2003).

Few studies examine ventilation rate in offices, although there is one comprehensive study by the EPD (1995). However, the average ventilation rate reported in this study is much smaller than in other studies and there are some significantly higher ventilation rates in the sample. The results of other office studies also do not show much uniformity given the large range in the volume of the tested rooms (EPD, 1995; Chao and Chan, 2001; Law et al., 2001). All this indicates that the ventilation rate of offices might have a large standard deviation depending on the office’s function, building year, customer requirements, etc.

The ventilation rate for other categories is hard to find in the literature. There are no

tracer gas tests of ventilation rate in schools in Hong Kong. The two studies that monitored CO_2 levels suggested that natural ventilation can increase ventilation rates in classrooms (Lee and Chang, 2000; Lee et al., 2002a). There is only one tracer gas experiment conducted in the sub-groups of shops and libraries, and it used a relatively small room volume. Ventilation rates tested in hospitals are mostly conducted in isolation rooms and operation rooms (Chow and Yang, 2003; Li et al., 2007). Consequently, there is a need for more data on the ventilation rates in large volume locations such as shopping malls, libraries, and hospital waiting rooms. Ventilation rates for high occupant density locations such as schools have also been largely neglected in the literature.

To summarize the observed data, the average ventilation rates in the above-mentioned sub-groups are listed in Table A19. In the disease dynamic model, we set the baseline scenario of the ventilation condition to be mechanical ventilation. As the ventilation rate might be underestimated in schools, we used 2 ACH instead of the 1.85 ACH given in the collected data. As the occupant density of all of the locations are set differently, the ventilation rates per person in the same type of locations are accordingly different. The distribution of ventilation rate per person in different places under the baseline ventilation scenario, calculated according to sizes of all locations, is plotted in Figure A4.

Table A19 Estimation of ventilation rates in different type of buildings

Name	Ventilation type	ACH
**Homes:**	Naturally ventilated with open windows	5
	Closed windows with air conditioner	0.7
	Closed windows without air conditioner	0.3
**Offices:**	Mechanical ventilation	1
**Schools:**	Nature ventilation with open windows and ceiling fans	16
	Mechanical ventilation	1.85
**Shops:**	Mechanical ventilation	1
**Libraries:**	Mechanical ventilation	1.4
**Public transportations**	Mechanical ventilation	4*

*As most buses in Hong Kong are double deckers, the ventilation rate is assumed to be smaller than the reference value.

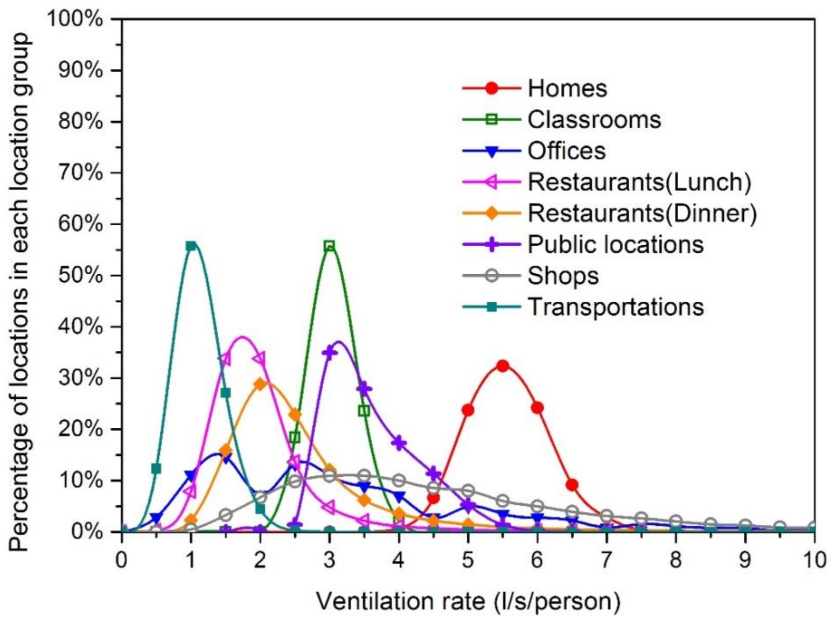

Figure A4 Ventilation rate (l/s/person) distribution in different types of locations. Air change rates are set as the referencing parameter, 0.7 ACH in homes, 2 ACH in classrooms, 1 ACH in offices, 1 ACH in restaurants (assumed value), 1.4 ACH in public places, 1 ACH in shops, and 4 ACH in transportation locations.

3. **Determining the quanta generation rate**

We assumed a complete susceptibility of the whole population. Outbreaks with three different quanta generation rates (1, 2, and 3 quanta/h) were simulated with our indoor social contact network transmission model. The results of the three cases are listed in Table A20 and Figure A5.

Table A20. Control methods for increasing ventilation rates.

Quanta generation rate, Q (quant/h)	Attack rate, δ	Average basic reproductive number, R_o	Time of peak infection, T_p (day)	Percentage of infectors on day T_p, λ_p
1	70.83%	2.22	303	9.59%
2	93.76%	4.03	154	24.90%
3	97.47%	5.83	113	32.70%

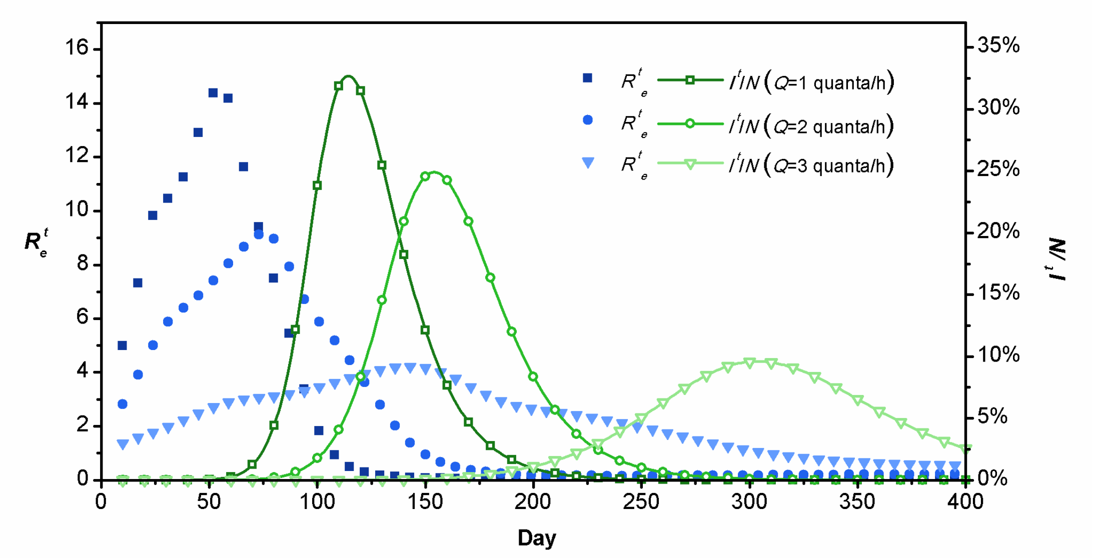

Figure A5. Reproductive numbers and percentages of infectors under quanta generation rates of 1, 3, and 5 quanta/hour

The effective reproductive number, R_o^t, is defined as the average number of secondary cases caused by one infector at time t in the epidemic (Haydon et al., 2003; Nishiura et al., 2006). In this model, the virus shedding abilities of infectors are assumed to be identical and constant across the infectious period. Hence, R_o^t can be calculated according to the expected number of infectors, I^t, and the expected number of new infectors and for day t as

$$R_e^t = (S^t - S^{t+1}) / (\gamma I^t) \qquad \text{(A5)}$$

S^t is the expected number of susceptible individuals on day t. The changes in R_o^t under

different quanta generation rates are plotted in Figure A5. The basic reproductive number on day t, R_o^t, can be calculated according to R_e^t as

$$R_o^t = R_t N / S^t \qquad \text{(A6)}$$

N is the population size. Variations of the basic reproductive number through time are caused by applying the probability model and the spread of infection through large hubs. The infection stages of each individual are represented by probabilities and the number of infectors is calculated as the expectation of infectors. At the beginning of the outbreak, for most individuals, the probability of being an infector is very small; using this small probability to calculate the probability of infecting others in some locations might underestimate the transmission. Furthermore, the location visiting behavior of an individual at a certain time in real life is in the model replaced by the probabilities of visiting several favorite locations; hence, there might be a dilution of the transmission ability of an infector in a simulated probability network in a single day, compared to a fixed network. Thus, although the reproductive number is small in the initial stage of the outbreak, the connectivity of the probability network is larger than that of the fixed network. Hence, the underestimation is eliminated when the disease becomes more widely spread.

A decrease in R_o^t is also observed during the simulated transmission period. This phenomenon is caused by the structure of the indoor social contact network. Infection tends to spread in and around large transmission hubs first and then be introduced to nodes far from large hubs. Hence, in the early stage of an outbreak, infectors attending large and crowed locations have a higher probability of infecting others. Once most of the individuals connected with large hubs are infected, the spreading trend is moderated. Therefore, if we adopt the average basic reproductive number calculated at the outbreak, there might be an overestimation or underestimation of the scale of the outbreak.

To address this issue, we calculated the average reproductive number from the day when 0.1% of the population is infected to the day when the attack rate reaches 90% of the final attack rate. The average basic reproductive numbers, shown in Table A20, approximately follow a linear correlation with quanta generation rates within the range of 1 ~ 3 quanta/h; this is consistent with the estimated and normally adopted basic reproductive numbers of smallpox transmission. Hence, we adopt these three cases, representing low, medium, and high transmission rate scenarios.

References

Cao, H. Z., Hardy, J. R., Douglass, R. W., Dawkins, P. T. and Dunbar S. R. (1992) Fractal character of two-dimensional fluid mixing at both continuum and atomic levels, *Phys Rev A*, 45, 3841-3844.

Census and Statistics Department HKGOV (2006) 2006 Population By-census, Main Report: Volume 1. Retrieved 05/12, 2009, from http://www.statistics.gov.hk/publication/stat_report/population/B11200472006XXXXB0400.pdf.

Census and Statistics Department HKGOV. (2007). Hong Kong in Figures. Retrieved 3/22, 2009, from http://www.censtatd.gov.hk/FileManager/EN/Common/hkinf.pdf.

Chan, A. T. (2003) Commuter exposure and indoor-outdoor relationships of carbon oxides in buses in Hong Kong, *Atmospheric Environment*, 37, 3809-3815.

Chan, E. H. W., Cheung, A. A. C. and Lee, G. K. L. (2006) Statutory control of indoor natural ventilation concerning radon level and health, *Structural Survey*, 24, 114-126.

Chan, M. Y. and Ho, H. K. (2005) Ventilation rate for improving indoor radon level, *Architectural Science Review*, 48, 329-336.

Chao, C. Y. and Chan, G. Y. (2001) Quantification of indoor VOCs in twenty mechanically ventilated buildings in Hong Kong, *Atmospheric Environment*, 35, 5895-5913.

Chao, C. Y. and Wong, K. K. (2002) Residential indoor PM10 and PM2.5 in Hong Kong and the elemental composition, *Atmospheric Environment*, 36, 265-277.

Chao, C. Y. H. (2001) Comparison between indoor and outdoor air contaminant levels in residential buildings from passive sampler study, *Building and Environment*, 36, 999-1007.

Chao, C. Y. H. and Tung, T. C. (2001) An empirical model for outdoor contaminant transmission into residential buildings and experimental verification, *Atmospheric Environment*, 35, 1585-1596.

Chao, C. Y. H., Tung, T. C. W. and Burnett, J. (1997) Influence of ventilation on indoor radon level, *Building and Environment*, 32, 527-534.

Chow, T. T. and Yang, X. Y. (2003) Performance of ventilation system in a non-standard operating room, *Building and Environment*, 38, 1401-1411.

Edmunds, W. J., O'Callaghan, C. J. and Nokes, D. J. (1997). Who mixes with whom? A method to determine the contact patterns of adults that may lead to the spread of airborne infections, *Proc Biol Sci*, 264, 949-957.

EPD (1995) *Consultancy Study for Indoor Air Pollution in Offices and Public Places in Hong Kong*, Hong Kong SAR, Environmental Protection Department.

Godish, T. and Spengler, J. D. (1996) Relationships between ventilation and indoor air quality: A review. *Indoor Air-International Journal of Indoor Air Quality and Climate*, 6, 135-145.

Haydon, D. T., Chase-Topping, M., Shaw, D. J., Matthews, L., Friar, J. K. et al. (2003) The construction and analysis of epidemic trees with reference to the 2001 UK foot-and-mouth outbreak. *Proceedings of the Royal Society B: Biological Sciences* 270, 121-127.

Ho, F. W. H. (2004) The Hong Kong population situation and its development trend. Retrieved 03/05, 2009, from http://www.censtatd.gov.hk/FileManager/EN/Content_248/the_hong_kong_population_situation_and_its_development_trend.pdf

Lam, K. S., Chan, E. H. W., Chan, D. W. T., Fung, W. Y., Law, K. C. and Tai, C. T. (2007) Minimum outdoor air supply for radon in high rise residential buildings - natural ventilation vs air-conditioning unit, *International Journal of Ventilation*, 5, 427-436.

Law, A. K. Y., Chau, C. K. and Chan, G. Y. S. (2001) Characteristics of bioaerosol profile in office buildings in Hong Kong, *Building and Environment*, 36, 527-541.

Lee, S. C. and Chang, M. (2000) Indoor and outdoor air quality investigation at schools in Hong Kong, *Chemosphere*, 41, 109-113.

Lee, S. C., Chang, M. and Chan, K. Y. (1999) Indoor and outdoor air quality investigation at six residential buildings in Hong Kong, *Environment International*, 25, 489-496.

Lee, S. C., Guo, H., Li, W. M. and Chan, L. Y. (2002a) Inter-comparison of air pollutant concentrations in different indoor environments in Hong Kong, *Atmospheric Environment*, 36, 1929-1940.

Lee, S. C., Li, W. M. and Ao, C. H. (2002b) Investigation of indoor air quality at residential homes in Hong Kong - case study, *Atmospheric Environment*, 36, 225-237.

Lee, S. C., Li, W. M. and Chan, L. Y. (2001) Indoor air quality at restaurants with different styles of cooking in metropolitan Hong Kong, *Science of the Total Environment*, 279, 181-193.

Lee, T. K. C. and Yu, K. N. (2000) Effects of air conditioning, dehumidification and natural ventilation on indoor concentrations of Rn-222 and Rn-220, *Journal of Environmental Radioactivity*, 47, 189-199.

Li, Y., Ching, W. H., Qian, H., Yuen, P. L., Seto, W. H., Kwan, J. K., Leung, J. K. C., Leung, M. and Yu, S. C. T. (2007) An evaluation of the ventilation performance of new SARS isolation wards in nine hospitals in Hong kong. *Indoor and Built Environment*, 16, 400-410.

Lin, Z. P. and Deng, S. M. (2003) The outdoor air ventilation rate in high-rise residences employing room air conditioners, *Building and Environment*, 38, 1389-1399.

Man, C. K. and Yeung, H. S. (1999) Modeling and measuring the indoor radon concentrations in high-rise buildings in Hong Kong, *Applied Radiation and Isotopes*, 50, 1131-1135.

Mossong, J., Hens, N., Jit, M., Beutels, P., Auranen, K., Mikolajczyk, R., Massari, M., Salmaso, S., Tomba, G. S., Wallinga, J., Heijne, J., Sadkowska-Todys, M., Rosinska, M. and Edmunds, W. J. (2008) Social contacts and mixing patterns relevant to the spread of infectious diseases, *PLoS Med*, 5, e74.

Mui, K. W. and Shek, K. W. (2005) Influence of in-tunnel environment to in-bus air quality and thermal condition in Hong Kong, *Science of the Total Environment*, 347, 163-174.

Nishiura, H., Schwehm, M., Kakehashi, M. and Eichner, M. (2006) Transmission potential of primary pneumonic plague: Time inhomogeneous evaluation based on historical documents of the transmission network. *Journal of Epidemiology and Community Health*, 60, 640-645.

Rim, D., Siegelm J., Spinhirne, J., Webb, A. and McDonald-Buller, E. (2008) Characteristics of cabin air quality in school buses in Central Texas, *Atmospheric Environment*, 42, 6453-6464.

Seppanen, O. A., Fisk, W. J. and Mendell, M. J. (1999) Association of ventilation rates and CO2 concentrations with health and other responses in commercial and institutional buildings, *Indoor Air-International Journal of Indoor Air Quality and Climate*, 9, 226-252.

Wallinga, J., Teunis P. and Kretzschmar, M. (2006) Using data on social contacts to estimate age-specific transmission parameters for respiratory-spread infectious agents, *Am J Epidemiol*, 164, 936-944.

Wan, K. S. Y. and Yik, F. H. W. (2004) Representative building design and internal load patterns for modelling energy use in residential buildings in Hong Kong, *Applied Energy*, 77, 69-85.

Wargocki, P., Wyon, D. P., Sundell, J., Clausen, G. and Fanger. P. O. (2000) The effects of outdoor air supply rate in an office on perceived air quality, Sick Building Syndrome (SBS) symptoms and productivity, *Indoor Air*, 10, 222-236.
